# Asymmetric nanocapsules via elongated liposome templated polymerization (ELTP) mediated by RAFT polymerization

**DOI:** 10.1007/s13346-025-01805-z

**Published:** 2025-03-06

**Authors:** Yunxin Xiao, Alexander W. Jackson, Angel Tan, John F. Quinn, Simon Crawford, Ben J. Boyd

**Affiliations:** 1 Drug Delivery, Disposition and Dynamics, Monash Institute of Pharmaceutical Sciences, Monash University Parkville Campus, 381 Royal Parade, Parkville, VIC 3052 Australia; 2 ARC Centre of Excellence in Convergent Bio-Nano Science and Technology, Monash University Parkville Campus, 381 Royal Parade, Parkville, VIC 3052 Australia; 3 Agency for Science, Technology and Research (A*STAR), 1 Pesek Road, Jurong Island, 627833 Singapore; 4 Department of Chemical Engineering, Faculty of Engineering, Monash University, Clayton, VIC 3800 Australia; 5 Ramaciotti Centre for Cryo-Electron Microscopy, Monash University Clayton Campus, 15 Innovation Walk, Clayton, VIC 3800 Australia; 6 Department of Pharmacy, University of Copenhagen, 2100 Copenhagen, Denmark

**Keywords:** Elongated nanocapsules, Morphology, Asymmetric liposomes, Ciprofloxacin-loaded nanocapsules, RAFT polymerization, ELTP, Drug delivery, Nanocrystals, Encapsulation

## Abstract

Polymeric nanocapsules comprised of hydrophobic shells and hollow aqueous interiors are an extremely useful class of nanomaterial, particularly in the encapsulation and controlled delivery of hydrophilic cargo. Generally prepared via droplet or latex templation approaches, polymeric nanocapsules are mostly spherical. Controlling the morphology of hollow nanocapsules is an intriguing design challenge. Non-spherical, or elongated, templates are often inorganic materials which do not directly impart a hollow interior, and their post-polymerization removal is not straightforward. This study outlines a novel strategy for the preparation of elongated nanocapsules, wherein elongated liposomes are deployed as hollow templates. Initially, ciprofloxacin drug nanocrystals were utilized to facilitate the formation of elongated liposomes, followed by adsorption of reversible addition-fragmentation chain transfer (RAFT) oligomers. Subsequent chain-extension polymerization furnished the desired elongated nanocapsule morphology. This proof-of-concept study contributes towards the goal of elongated nanocapsule synthesis, a morphology which can impart improved circulation times in the field of drug delivery.

**Graphical Abstract:**

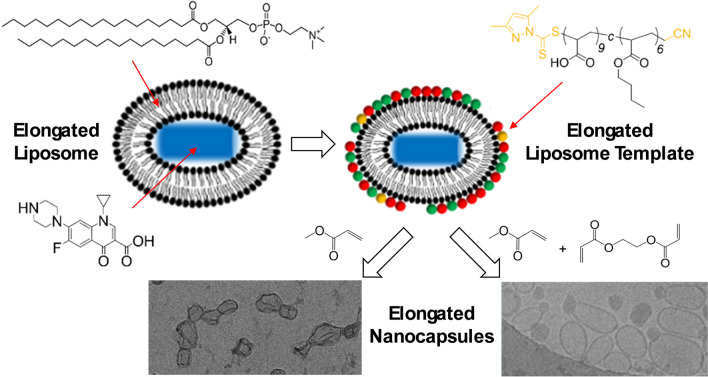

**Supplementary Information:**

The online version contains supplementary material available at 10.1007/s13346-025-01805-z.

## Introduction

Polymeric nanocapsules offer a versatile platform in the fields of consumer goods, coatings, agrochemicals and nanomedicine, owing to their diverse functionality, chemical robustness and high accessibility [1–3]. In the field of drug delivery, carriers with cross-linked polymeric exteriors can yield improved retention and controlled release, when compared to non-covalently self-assembled nanoparticles such as polymeric micelles and liposomes [4, 5]. Hollow polymeric nanocapsules are frequently prepared using a template. Sacrificial hard templates include gold nanoparticles [6], ellipsoidal Fe_2_O_3_ nanocrystals [7] and SiO_2_ particles [8, 9]. Soft templates, which do not require post-polymerization removal, have been used to prepare spherical and non-spherical polymeric nanocapsules. These have included dimethyldioctadecylammonium bromide (DODAB) vesicles [10–14], sodium dodecylbenzenesulfonate (SDBS) vesicles [15] and liposomes made of egg phosphatidylcholine (egg-PC) [16]. Vesicle templating methods [10–12, 17, 18] have been used to prepare nanocapsules with various morphologies, providing control over the chemical composition, physical properties and shell thickness. For drug delivery purposes, soft templates with hollow interiors provide the added opportunity to pre-encapsulate pharmaceutical actives.

The relationship between nanoparticle geometry and cellular interactions has been widely investigated with a drive towards decreasing internalization of drug delivery carriers by macrophages of the immune system [19–21]. Non-spherical drug delivery particles have shown decreased accumulation in liver and longer circulation times compared to spherical particles [22]. Elongated nanoparticles demonstrated to have this desirable behaviour include cylindrical filomicelles made from amphiphilic block copolymers [23], elliptical disk shaped polystyrene particles [24, 25] and cross-linked poly(ethylene–glycol) hydrogels of varying shape [26]. Drug delivery systems with non-spherical geometry possess greater probability of reaching the site of action and thus improving the delivery efficiency.

**Fig. 1:**
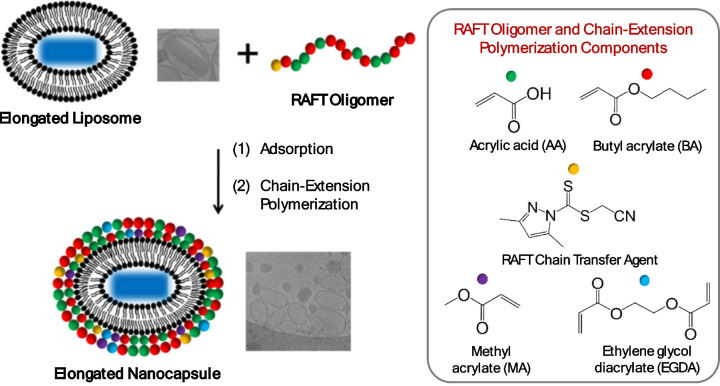
Illustration of elongated nanocapsule formation via elongated liposome templated polymerization (ELTP). After elongated liposome formation, RAFT oligomers (comprised of acrylic acid and butyl acrylate) are adsorbed onto the template surface, followed by chain-extension polymerization with methyl acrylate (MA) and ethylene glycol diacrylate (EGDA)

Although polymeric nanocapsules have been previously prepared, there remains a need for a tunable, scalable and effective methodology for the preparation of elongated nanocapsules. While inorganic templates can be used to obtain the elongated morphology, the use of strong solvents or high temperature during template removal renders pharmaceutical loading non-trivial [27–29]. Soft templates such as lipid or surfactant vesicles including DODAB, SDBS or liposomes made of egg-PC were also investigated to form non-spherical polymeric nanocapsules [30], but were not amenable to drug loading using the typical pH or ion gradient approaches. A recent review summarizes the various types of non-spherical nanocapsules and their formation process [31]. Herein, elongated nanocapsules are prepared via elongated liposome templated polymerization (ELTP); this novel pathway is illustrated in Fig. 1. Elongated liposomes were prepared via the growth of rod-shaped ciprofloxacin drug nanocrystals inside the liposome template [32]. Ciprofloxacin drug nanocrystals are formed by freeze-thawing drug-loaded liposomes, which have previously been used as a high-payload pulmonary inhalation treatment to decrease the dosage frequency for patients with cystic fibrosis [33, 34]. Some other drugs, such as some chemotherapy drugs, doxorubicin [35] and paclitaxel, are also able to form drug nanocrystals inside liposomes even without freeze-thawing. The concept of crystallized drugs inside drug delivery vesicles is highly relevant in the context of drug delivery to increase drug payload and achieve a sustained release [31]. After the formation of the elongated liposome template, RAFT oligomers were adsorbed onto the surface of liposomes. This process was characterized by zeta potential measurements and cryogenic-transmission electron microscopy (cryo-TEM). Subsequently, chain-extension polymerization was monitored using gas-chromatography mass-spectrometry (GCMS). After polymerization, the formation of hollow nanocapsules further confirmed successful adsorption and polymerization on the liposome surface, as opposed to in the aqueous phase which results in solid nanoparticles. The stability of the elongated nanocapsules was confirmed in the present of Triton X-100, confirming the formation of a cross-linked polymeric shell. Upon successful polymerization the size distribution of nanocapsules was in the 100–200 nm range. In the absence of polymer, complete lysis of the lipid bilayer led to the presence of micelles alone (approximately 10 nm). The effects of using ethylene glycol diacrylate (EGDA) as the crosslinker and two different monomer addition profiles (bulk addition and slow addition) on the morphology of nanocapsules were compared. Approximately 10–20% of the nanocapsules prepared with EGDA via the bulk addition method remained elongated even after the disappearance of drug nanocrystals.

The motivation behind this work was that the application of these drug crystal-loaded liposomes as polymerization templates could not only afford the elongated morphology, encapsulate pharmaceutical actives that are sensitive to hard templates or difficult to be encapsulated within surfactant vesicles, but also present the possibility to vary the aspect ratio of the final nanocapsules by modifying the lipid compositions in the liposomal formulation [36, 37]. The stability of the drug nanocrystals after the adsorption and polymerization process confirms the feasibility of using such a template to prepare elongated nanocapsules with potential application as a high drug-payload, long-circulating and controlled release drug delivery system.

## Experimental Section

### Materials

Hydrogenated soy phosphatidylcholine (HSPC) was purchased from Avanti Polar Lipids, Inc. (Alabaster, AL, USA). Ammonium sulfate (99.0% purity), potassium chloride (99% purity), sucrose (99.5% purity), cholesterol (> 99% grade), Triton X-100 (BioXtra), methyl acrylate (MA) (90% purity) and ethylene glycol diacrylate (EGDA) (99% purity) were purchased from Sigma-Aldrich (St. Louis, MO, USA). Ciprofloxacin monohydrate hydrochloride was purchased from TCI (Japan). Sodium chloride (99.7% purity) and disodium hydrogen orthophosphate (99% purity) were purchased from Chemsupply Pty Ltd (Gilman, SA, Australia). Potassium dihydrogen phosphate (99% purity) was purchased from Ajax Finechem (NSW, Australia). Nucleopore track-etch extrusion membranes were purchased from Whatman (Maidstone, Kent, UK). Amicon ultrafiltration cells and ultrafiltration discs were purchased from Merck Millipore (Burlington, Massachusetts, USA). 4,4'-Azobis(4-cyanopentanoic acid) (ACPA, V-501) was purchased from Wako Chemical. Water was from a Milli-Q system (Millipore, Sydney, Australia).

### Preparation of elongated liposomes

**Fig. 2:**
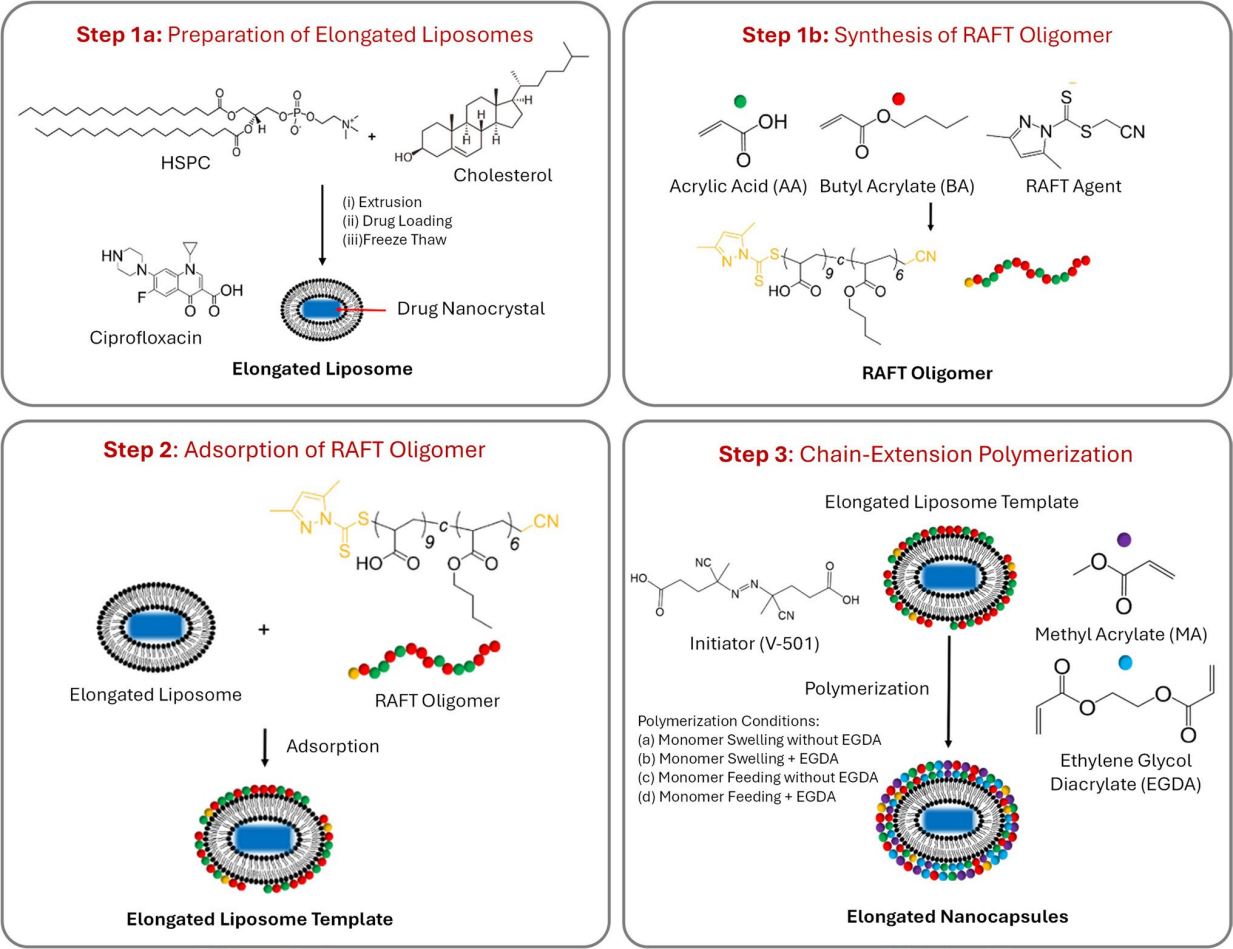
Schematic of three steps undertaken towards preparation of elongated nanocapsules. **Step 1a** – Preparation of elongated liposomes encapsulating ciprofloxacin drug nanocrystals. Step 1b – Synthesis of RAFT oligomer. **Step 2** – Adsorption of RAFT oligomers on elongated liposomes. **Step 3** – Polymerization to form elongated nanocapsules. During Step 3, four different nanocapsule polymerization conditions are investigated (a) monomer swelling without EGDA cross-linker, (b) monomer swelling with EGDA cross-linker, (c) monomer feeding without EGDA cross-linker and (d) monomer feeding with EGDA cross-linker

Liposomal templates were prepared via the thin-film hydration method (Fig. 2, Step 1a). For a 250 mg formulation, 175 mg of HSPC and 75 mg of cholesterol were dissolved in chloroform, and the solvent removed by evaporation to obtain a homogeneous lipid film. Most of the solvent was removed with an initial pressure of 400 mbar using a Buchi vacuum controller V-805 connected to a Buchi Rotavapor R-205 rotatory evaporator in a 40 °C water bath, followed by further evaporation at continuous mode for one hour to remove the residual solvent. The lipid film was then hydrated with 2.5 mL of 500 mM ammonium sulfate solution (pH 3.0) to yield a 10 wt% lipid composition. Unilamellar vesicles were then produced by passing the hydrated lipid through a mini extruder (21 times) with double-stacked Nuclepore track-etch membranes with a pore sizes of 1000 nm and then 200 nm. Then ultrafiltration was performed to exchange the external buffer to PBS (pH 7.4) using Ultracel^®^ regenerated cellulose ultrafiltration disc of 100 kDa in an Amicon ultrafiltration cell. PBS buffer (22.5 mL) was added to every 2.5 mL of extruded liposomes (250 mg lipid formulation at 10 wt%) for buffer exchange and 2.5 mL of ultrafiltered formulation was collected at the end. To perform drug loading, ciprofloxacin hydrochloride monohydrate (367.8 g/mol) was dissolved in water at a concentration of 20 mg/mL. An equal volume of this solution (2.5 mL) was added to the ultrafiltered liposomes (2.5 mL). The resulting solution was vortexed and incubated at 70 °C for 1 h. Dilution was then performed by adding 20 mL of PBS buffer to 5 mL of drug-loaded formulation. Sucrose was then added as a cryogenic protectant for the freeze-thaw process. Sucrose was dissolved in water at a concentration of 100 mg/mL then equal volume (25 mL) was added to the dilute formulation (25 mL) to give a final sucrose concentration of 50 mg/mL. Ciprofloxacin drug nanocrystals were formed by freezing the samples in liquid nitrogen and thawing at room temperature. Ciprofloxacin nanocrystals were formed during the thawing cycle of the freeze–thaw process, which stretched the liposomes to an elongated shape [32].

### Adsorption of RAFT oligomers onto empty and elongated liposomes

The RAFT oligomer, oligo(acrylic acid-co-butyl acrylate), was then adsorbed on the surface of spherical empty liposomes and elongated drug-loaded liposomes. RAFT oligomer (Fig. 2, Step 1b) was synthesized at the Institute of Sustainability for Chemicals, Energy and Environment (ISCE) and the synthetic method was reported previously by Rusli et al. [12]. The oligomer was characterized by size exclusion chromatography (SEC) and nuclear magnetic resonance (NMR) (Supplementary Information 1). The adsorption method previously described in the work of Rusli et al., utilized positively-charged vesicles made with dioctadecyldimethylammonium bromide (DODAB) to attract the negatively charged RAFT oligomer. The amount of RAFT oligomer and corresponding stability of adsorbed vesicles were quantified to validate the adsorption method [12]. In this work, the hydrophobicity of the RAFT oligomer was used to drive the adsorption onto the liposome surface. For all adsorption experiments, the molar ratio of RAFT oligomer to the main lipid in the formulation, HSPC, was kept at 1:2. When performing the adsorption to empty liposomes, in order to keep the stoichiometry the same between RAFT oligomers and lipid as the adsorption experiment for elongated liposomes, 1 mL of water was added to the 1 mL of ultrafiltered empty liposomes to mimic the drug loading process, followed by a dilution with 8 mL of PBS buffer. For a 100 mg batch of empty liposomes with 70 mg of HSPC, 75 mg of RAFT oligomer was used to make up 10 mL of solution with PBS buffer. Sodium hydroxide solution was added to assist the dissolution of RAFT oligomer and the final pH was adjusted to 7.4. Then 5 mL of RAFT oligomer solution was placed in a round-bottom flask, then, upon stirring, 5 mL of dilute empty liposomes were added with a syringe driver at a rate of 0.5 mL/min at room temperature. With the elongated freeze-thawed liposomes, the addition of sucrose solution diluted the elongated formulation by half, therefore for a 250 mg batch with 175 mg of HSPC, 187.5 mg of RAFT oligomer was used to make up 50 mL of solution with PBS buffer. After adjusting the final pH to 7.4, 50 mL of RAFT oligomer solution was placed in a round-bottom flask, upon stirring, 50 mL of elongated liposomes were added with a syringe driver at a rate of 0.5 mL/min (Fig. 2, Step 2). Adsorbed empty and elongated liposomes were stirred for another hour after complete addition and vitrified for cryo-TEM imaging.

### Chain extension and cross-linking via RAFT polymerization

RAFT polymerization was performed to convert the elongated liposomes with adsorbed RAFT oligomer into intact elongated polymer-coated nanocapsules (Fig. 2, Step 3). Two factors were explored to investigate the relationship between reaction kinetics and the final morphology of the elongated nanocapsules; the presence or absence of the cross-linker and the method of monomer addition. The absence of cross-linker has been linked to the growth of an extra solid polymeric particle attached to the main nanocapsules [12]. Two methods of addition, either adding the monomer at once (bulk addition method) or gradually through a syringe driver at a constant speed (slow addition method), might alter the reaction kinetics and thus the thickness or the morphology of the final nanocapsules. For a 250 mg lipid formulation (0.223 mmol HSPC, 0.115 mmol RAFT oligomer), 0.056 mmol of 4,4'-azobis(4-cyanopentanoic acid) (V-501, 10-h half-life temperature of 69 °C) were dissolved in 1 mL PBS buffer to keep the molar ratio between RAFT oligomer and the initiator to be 2:1. Dissolution of V-501 was assisted by adding NaOH solution and pH was adjusted to 7.4. V-501 was used as a water-soluble initiator to assist the polymerization reaction occurred in the interface of water and monomer. Methyl acrylate (MA) was used as the monomer and ethylene glycol diacrylate (EGDA) was used as the cross-linker for all experiments. Previous research showed that using methyl acrylate as the main monomer instead of methyl methacrylate ensured the formation of a homogenous layer of nanocapsule shells, due to its capability to chain-extend the initial RAFT oligomers [12]. The molar stoichiometry of MA:HSPC was 46:1 according to previous work on spherical nanocapsules [12]. For experiments without a cross-linker, 0.93 mL of MA (0.95 g/cm^3^, 880 mg, 86.09 g/mol, 10.2 mmol) was used. For experiments with EGDA as the cross-linker, MA and EGDA were pre-mixed prior to polymerizations with a weight ratio of 10:1. The stoichiometry (Table 1) was adapted from the optimized method of RAFT polymerization to prepare spherical nanocapsules reported by Rusli et al. [12].

**Table 1:** Stoichiometry of components used to prepare elongated nanocapsules

Formulation	Component(s)	Mass (mg)	*M*_r_ (g/mol)	Mole (mmol)	Density (g/cm^3^)	Process	Volume (mL)		Eq.^*b*^
							**Addition**	**Total**^***a***^	
Elongated Liposomes	HSPC	175	783.7	0.223	-	Thin film hydration	2.5	2.5	1
	Cholesterol	75	386.7	0.194	-				0.87
	Ciprofloxacin	50	385.8	0.130	-	Drug loading	2.5	5	0.58
	PBS Buffer	-	-	-	-	Dilution	20	25	-
	Sucrose	2500	342.3	7.304	-	Freeze–thaw	25	50	33
Adsorbed Liposomes	RAFT Oligomer	187.5	1629	0.115	-	Adsorption	50	100	0.5
Elongated Liposomes	V-501	15.6	280.28	0.056	-	Polymerization	1	101	0.25
	MA	880	86.09	10.22	0.95		0.92	102	46
	EGDA	88	170.16	0.517	1.05		0.08		2.3

^*a*^Cumulative volume after each addition and ^*b*^ molar equivalence relative to HSPC

After adsorption of the oligomer, elongated liposomes were placed in a round-bottom flask and de-oxygenated for 20 min with N_2_ before polymerization. Monomer MA, monomer mixture of MA and EGDA, and solutions of initiators were not de-oxygenated due to the small volume compared to the 100 mL of liposomes with adsorbed oligomer. Monomers were either added, upon stirring, via the bulk addition method or the slow addition method at a rate of 0.5 mL/h with a syringe driver. Then the round-bottom flask was heated up to 70 °C for addition of the initiator, V-501. Reactions were left overnight for 18 h and samples were taken at the start and the end of reactions for characterization. The reaction was stopped by cooling the flask to room temperature and allowing air to diffuse into the flask. A summary of the preparation process and various samples made with different polymerization parameters is shown in Fig. 2. The progress of polymerizations were monitored by determining the concentrations of the residual methyl acrylate (MA) in the reaction flask, a volatile compound with a boiling point of 80 °C, using gas chromatography-mass spectrometry (GC–MS). A standard curve of MA was generated with MA in water at concentrations below 10 µg/mL (Supplementary Information 2). Samples (10 µL) were taken from the start and the end of the polymerizations and transferred to a headspace sample container and injected with a headspace injector (liquid injection was not practical for these samples with the presence of drug nanocrystals, highly conjugated polymer structures and high concentrations of salt in the buffer solution). All measurements were carried out using a Shimadzu GC-2010 Plus GC coupled with a HS-20 headspace sampler and TQ8050 mass spectrometer (MS) (Shimadzu, Kyoto, Japan). An Agilent DB-SELECT 624 UI column (30 m × 0.25 mm × 1.4 µm) was used with helium as the carrier gas at a pressure of 100 kPa, creating a flow rate of 1.72 mL/min and a linear velocity of 47.2 cm/sec. Initial column temperature was 40 °C and held for 2 min, then increased to 120 °C at a rate of 10 °C/min with 1-min holding time. The headspace sample vial was pressurised at 50 kPa gas pressure by the headspace sampler with an oven temperature of 120 °C, sample line temperature of 150 °C, and a transfer line temperature of 150 °C, an equilibrating time of 5 min, pressurising time of 1 min, pressure equilibrating time of 0.2 min, load time of 1 min, load equilibrating time of 0.2 min, injection time of 1 min, needle flush time of 5 min. The vapour was then injected via a direct injection mode. The mass spectrometer was operated with an ion source temperature of 230 °C, interface temperature of 250 °C, and a solvent cut time of 4 min. Under this condition, methyl acrylate had a retention time of 4.85 min and its mass spectrum was used to match with the in-built library for identification (Supplementary Information 3).

### Measurements of size distribution and zeta potential by dynamic light scattering (DLS)

Measurements of size distribution and zeta potential were performed for elongated liposomes, liposomes with adsorbed oligomer and polymerized nanocapsules using a Zetasizer Nano ZS DLS instrument from Malvern Instruments (Worcestershire, UK). The instrument uses a 4 mW He–Ne laser (λ = 632.8 nm) with a backscatter detection angle of 173°. The thermostatted sample chamber was set to 25 °C. The viscosity and refractive index of water at 25 °C (0.8937 cP and 1.333 respectively) were used as the dispersant for all measurements. Measurement position and attenuation was optimized by the instrument. A mean size (Z-ave) was determined in a standard cumulant analysis of the software whereas a general purpose (normal distribution) algorithm from the software was used for size distribution analysis. Data presented is all in intensity (%) distributions. The absorption and refractive index of lipid at the same temperature (0.01 and 1.45 respectively) were used as the material parameters for empty liposomes, elongated liposomes and liposomes with adsorbed oligomer. For polymerized nanocapsules, the absorption and refractive index of polystyrene (0.01 and 1.59 respectively) were used as the material parameters. The intensity particle size distribution (PSD) and zeta potential were quoted.

### Visualization with Cryo-TEM

The morphology of the empty liposomes with adsorbed oligomer, elongated liposomes and polymerized nanocapsules were visualized with cryogenic-transmission electronic microscopy (cryo-TEM), with freeze-thawed liposomes encapsulating drug nanocrystals as the control. Samples before and after the breakage with Triton X-100 (described below) were also imaged. A QUANTIFOIL R 1.2/1.3 copper 200 grid was glow discharged with a PELCO easiGlow discharger at 0.22 mbar for 45 s prior to sample loading. Samples (3.5 µL) were loaded using a Vitrobot FEI with a blot time of 2 s, a wait time of 0 s, a drain time of 0 s, a blot force of −3, a temperature of 20 °C and a humidity of 100%. A Gatan cryogenic transfer holder was used to transfer the sample and an FEI Tecnai 12 module electronic microscope was operated at 120 kV. The thickness of the shell was measured using ImageJ software. Since the shell was not sufficiently thick for a direct measurement, to minimise measuring errors, the full length of the major axis (of elongated nanoparticles) across the whole nanoparticle including the thickness of the shell, L1, were measured first. Then the inner length of the same axis excluding the thickness of the shell, L2, were measured (Supplementary Information 4). The thickness of the shell was then calculated by (L1-L2)/2. The experiments using DODAB vesicles as the template to prepare spherical nanocapsules, reported by Rusli et al. [12] were repeated and served as a comparison to evaluate the efficiency of the adsorption process on the novel elongated liposomes. Empty and elongated liposomes before and after adsorption were measured. For each sample, five individual nanoparticles with various sizes were chosen to calculate the layer thickness. All statistical analyses were completed with the GraphPad Prism (version 7.00) for Windows, GraphPad Software, La Jolla California USA, www.graphpad.com.

### Verification of polymeric shell via removal of liposome template after polymerization

The existence of a polymeric layer on the surface of the liposome templates was first investigated by destroying the liposome template by addition of excess Triton X-100 after polymerization. Triton X-100 is a non-ionic surfactant with a hydrophilic polyethylene oxide chain, which is used widely in lysis of biological or simulated membranes to extract cellular organelles or to rupture vesicles such as liposomes. Herein, the hypothesis was that the polymeric shell, especially the sample cross-linked with EGDA, would remain intact upon treatment with Triton X-100 solutions, while the bilayer of the liposome templates would be destroyed with Triton X-100. If the polymeric shell successfully formed on the surface of liposomes, a size distribution peak at about 150 nm would be present demonstrating the existence of the polymeric layer. If the polymeric shell did not form, Triton X-100 would be able to disrupt the vesicles and so only a micellar peak formed by Triton X-100 will be observed. Triton X-100 solutions were prepared in water at a concentration of 10 wt%. The freeze-thawed elongated liposomes as the control were diluted by half to match the concentration of the polymerized nanocapsules prior to the addition of Triton X-100 solutions. Triton X-100 solutions (1 mL) at different concentrations were added to each of the samples, followed by stirring for 1 h before testing the size distribution by DLS.

## Results and discussion

Ciprofloxacin was chosen as the model drug compound due to its ability to form drug nanocrystals within liposomes by the freeze–thaw methodology, providing the desired elongated shape [33, 36]. Ciprofloxacin was loaded within HSPC liposomes via the pH gradient approach, by setting the internal pH of liposomes to be 3.0. After drug loading, ciprofloxacin was then crystallized inside the liposomes which elongates the otherwise spherical liposomes (Fig. 2 Step 1a). A number of anti-cancer drugs are also able to form nanocrystals within liposomes [38]. However, their cytotoxicity prevents them from being useful as a templating agent for non-cancer applications and introduces additional safety concenrs on handling. Therefore, the antibiotic ciprofloxacin was used as the model drug throughout this proof-of-concept study. RAFT oligomers (Fig. 2 Step 1b) were then adsorbed onto the elongated liposome exteriors affording the elongated liposome templates (Fig. 2 Step 2). Subsequent chain-extension polymerization yielded elongated polymeric nanocapsules (Fig. 2 Step 3). RAFT polymerization was chosen as the polymerization technique due to its compatibility in aqueous media, and the reversible-deactivation radical polymerization mechanism facilitates chain-extension and the formation of a uniform polymeric layer. Four different chain-extension polymerization approaches were performed to investigate the impact of two factors on the morphology of the resulting nanocapsules. The use of a short-chain cross-linker, ethylene glycol diacrylate (EGDA), and the monomer addition profile. Monomer swelling (addition of all monomers at once to the reaction flask) and monomer feeding (addition of monomers to the reaction flask at a speed of 2 mL/h with a syringe driver) were the two addition processes investigated.

### Size distribution and zeta potential (DLS)

**Fig. 3:**
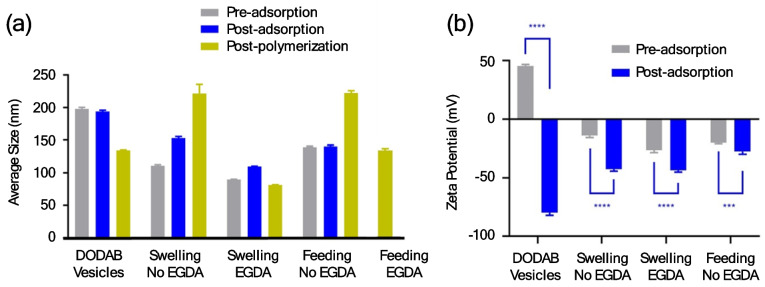
(**a**) Average size measured by DLS showed successful nanoconfinement provided by the 200 nm membrane-extruded liposome templates. The same batch of adsorbed liposomes was used to perform both feeding addition polymerization reactions. The polydispersity index (PDI) for DODAB vesicles pre-/post-adsorption and post-polymerization were 0.139, 0.127 and 0.162. Nanocapsules prepared via swelling addition without EGDA had PDIs of 0.187, 0.208, and 0.292, while nanocapsules prepared via swelling addition with EGDA had PDIs of 0.162, 0.118, and 0.112. Nanocapsules prepared via feeding addition with EGDA had PDIs of 0.143, 0.160, and 0.350. Nanocapsules prepared via feeding addition with EGDA post-polymerization had PDI of 0.348. (**b**) Zeta potential measurements for DODAB and liposome systems. ***P < 0.001 ****P < 0.0001 (t-test; two-tailed). Error bars represent the standard deviation (SD) for all samples (n = 4 for the size distribution of adsorbed DODAB vesicles and n = 5 for all other samples)

DODAB vesicles, which form spherical nanocapsules, were used as a comparative template system. The average size of DODAB and elongated liposome templates pre-/post-adsorption and post-polymerization with four different polymerization conditions are shown in Fig. 3a. The two experiments using monomer feeding addition were prepared with the same batch of elongated liposome templates, as such one set of data before polymerization is presented. For the DODAB system, as repeated according to Rusli et al*. *[12], the pre-adsorption templates were empty spherical DODAB vesicles. For the liposome systems, the pre-adsorption templates were elongated freeze-thawed liposomes encapsulating ciprofloxacin drug nanocrystals. The size of liposomes fell within the expected range, mainly as a result of using 200 nm extrusion membranes on the pre-crystallized liposomes that provided a nanoconfinement to control the size of the ciprofloxacin nanocrystals [36]. Elongated nanocapsules prepared in the presence of EGDA showed a slight decrease in average size after polymerization which might be due to the polymeric shell being more compact after chain-extension and cross-linking. It is important to note that in the algorithm of DLS, nanoparticles are assumed to have a spherical shape. Hence, size measurement by DLS for elongated nanoparticles are less accurate than that of spherical nanoparticles. Therefore, DLS measurement is only used as a general reference here. Detailed morphological and dimensional properties of nanocapsules such as the length of the major and minor axis were further investigated using cryo-TEM. Zeta potential measurements by DLS were used to investigate the adsorption of RAFT oligomers onto the surface of liposome templates compared to that of DODAB vesicles (Fig. 3b). DODAB vesicles possess positively charged surfaces. After adsorption of the negatively charged RAFT oligomers, the surface charge of the vesicles was inverted. For liposome templates, due to the ionization of the phosphate group in PBS buffer, a slightly negative zeta potential was observed pre-adsorption. After adsorption, the surface appeared to be more negative, but the change was less apparent than that of the DODAB vesicle system.

### Adsorption of RAFT oligomers onto empty and elongated liposomes

**Fig. 4:**
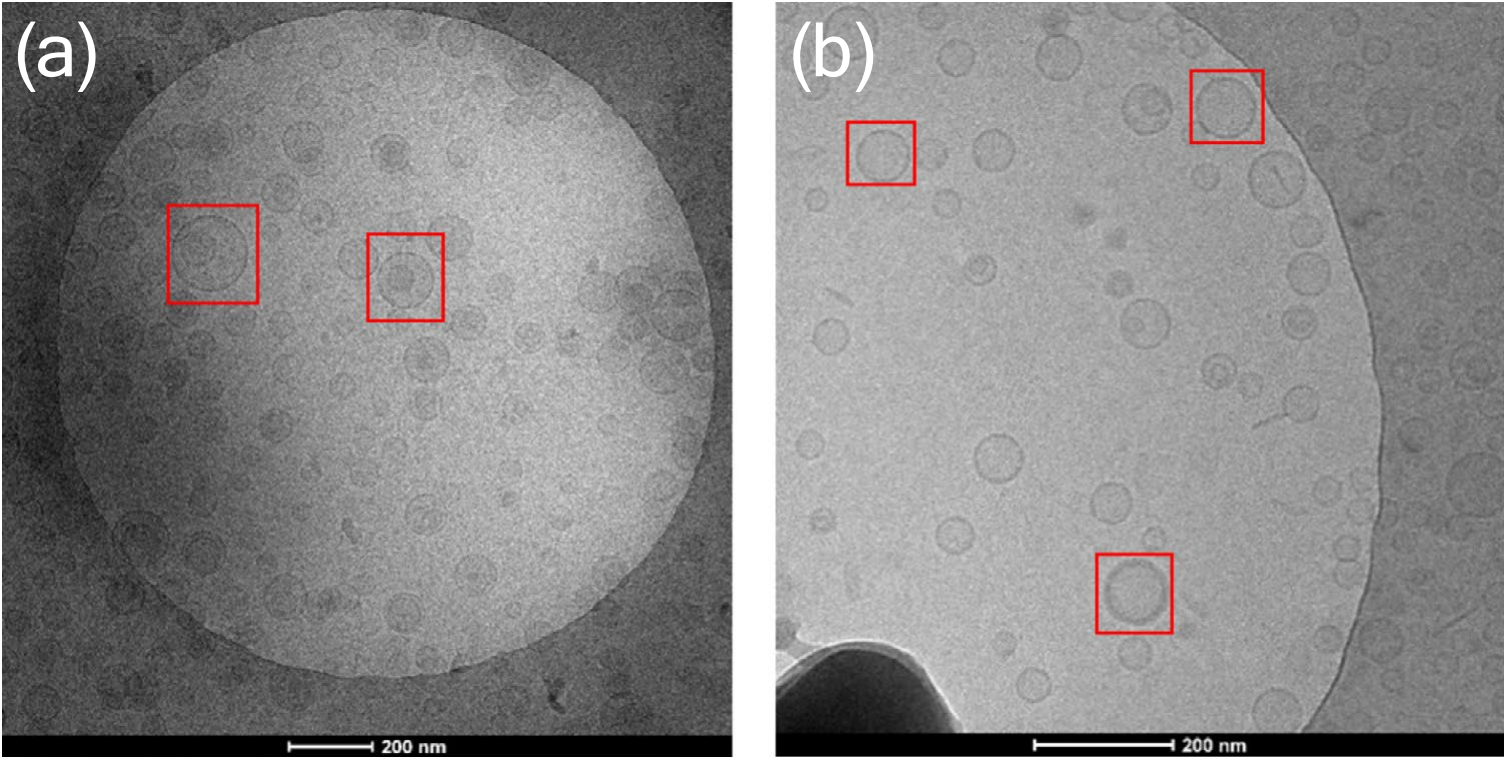
(**a**) Empty liposomes served as the control to observe the thickness of the lipid bilayer before adsorption, (**b**) empty liposomes with RAFT oligomers adsorbed onto the surface appeared to have thicker shells under cryo-TEM, indicating a successful adsorption. Circled liposomes are highlighted for more clear visual comparison

Spherical liposomes, without drug loading or freeze-thawing, were initially exposed to RAFT oligomers and the adsorption was monitored via cryo-TEM. Comparing the cryo-TEM micrographs of empty liposomes (Fig. 4a) [32], the thickness of the shell after adsorption (Fig. 4b) appeared to be greater suggesting a successful adsorption of the RAFT oligomers on the surface. However, compared to the DODAB vesicle templates that possess positively-charged membranes for adsorption [12], the increment of the shell thickness using neutral liposomes was less significant, judged by the visual appearance in the micrographs and a quantitative analysis using FIJI software to measure the thickness of the wall (Fig. 9).

**Fig. 5:**
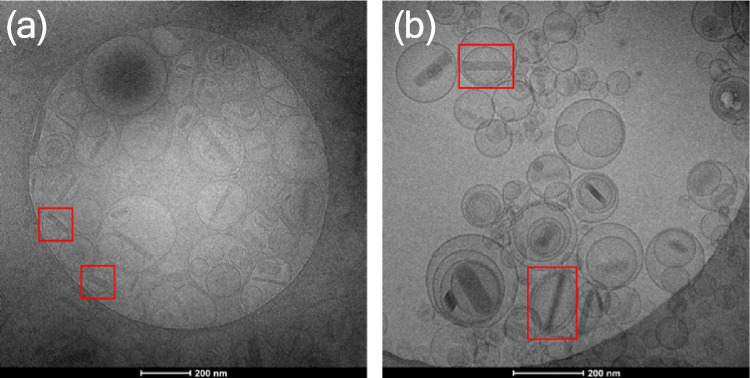
(**a**) Nanocrystals formed inside the liposomes after freeze-thawing and the formation of elongated liposomes. (**b**) Nanocrystals remained inside the liposomes post-adsorption of the RAFT oligomer, and the shell appeared to be slightly thicker than pre-adsorption. Several liposomes with intact drug nanocrystals are highlighted

RAFT oligomers were then adsorbed onto elongated liposomes containing ciprofloxacin drug nanocrystals to explore the possibility of using elongated liposomes as the template to prepare novel elongated nanocapsules. For liposomes before adsorption (Fig. 5a), with an encapsulation efficiency of over 90%, no drug nanocrystals appeared to form outside the liposomes. The encapsulation efficiency was determined by size exclusion chromatography and 90% of the added drug was encapsulated within liposomes. However, not all liposomes were occupied with drug nanocrystals indicating that the amount of drug added could potentially be further increased in future studies. The colloidal stability of the samples was maintained after adsorption (Fig. 5b) and no phase separation was observed, presumably due to the balance of hydrophobicity provided by the butyl chain and hydrophilicity provided by the carboxyl group in the RAFT oligomers, as this was first considered when the RAFT oligomer was designed [12]. Ciprofloxacin nanocrystals also remained inside the liposomes, indicating that the adsorption process did not disrupt the lipid bilayer or the nanocrystals. As was the case when the RAFT oligomer was adsorbed onto empty spherical liposomes, the thickness of the shell appeared to increase after adsorption, judging by their visual appearance, but the increment was again not as significant as that of the adsorption onto DODAB vesicles. The percentage of elongated adsorbed liposomes were relatively low, with empty liposomes and spherical liposomes containing drug nanocrystals present.

### Confirmation of chain-extension reaction

**Fig. 6:**
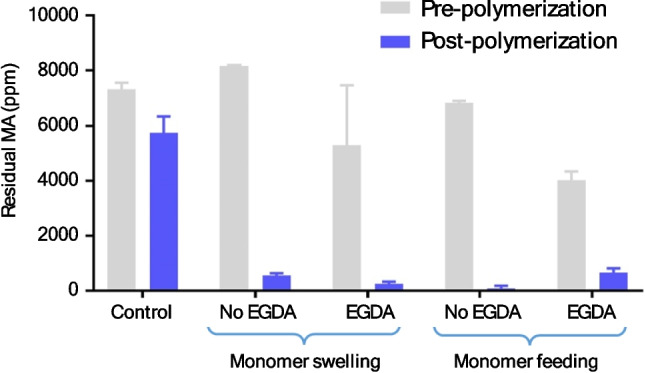
Consumption of MA in the four polymerization reactions (calculated via GC–MS). Monomer swelling without EGDA (93.1% complete, n = 2), monomer swelling with EGDA (95.3%, n = 3), monomer feeding without EGDA (98.7%, n = 2) and monomer feeding with EGDA (83%, n = 2 pre-polymerization and n = 3 post-polymerization). N refers to the number of samples taken for each timepoint

Gas chromatography-mass spectrometry (GC–MS) was used to confirm the extent of chain-extension polymerization reactions by determining the concentration of residual monomer, methyl acrylate (MA) (Fig. 6). For all reactions, over 80% MA was consumed, indicating that the reactions proceeded successfully. The absolute concentrations of MA pre- and post-polymerization was not quoted due to the high volatility of MA at room temperature. A control experiment was performed by addition of all reagents except for the initiator (V-501). No polymerization occurred in the control due to the lack of a free radical source and 18.2% of MA loss was detected, which reflected the detection error by GC–MS and potential evaporation of MA throughout the experiment. Detection errors might come from evaporation of MA in sample transfer, dilution and packing of the headspace vials. The pressurising step in the headspace injector might also create an un-even vapour distribution in the vial, resulting in different absolute amount of MA being injected to the GC column. This loss of MA in the control experiment is significantly lower than the > 80% observed in the presence of the initiator, confirming MA loss was due to polymerization for experimental groups. This analysis served as a successful stand-in as ^1^H NMR is not always suitable for heterogenous and cross-linked systems. The correlation between the amount of MA consumed and if they grow onto the surface of the liposome is elaborated in the next section with cryo-TEM.

### Effect of cross-linker (EGDA) on nanocapsule morphology

Four polymerizations were performed to investigate the impact of two factors on nanocapsule morphology. The use of a cross-linker, ethylene glycol diacrylate (EGDA), and the method of monomer addition. Since the RAFT oligomer is comprised of butyl acrylate and acrylic acid repeat units, methyl acrylate was chosen as the monomer to facilitate efficient chain-extension of the adsorbed RAFT oligomers, in turn ensuring formation of a homogeneous polymeric shell. Two different monomer addition methods were explored, swelling where monomers were added all at once to the reaction flask, and feeding where monomers were added to the reaction flask at a speed of 2 mL/h with a syringe driver.

**Fig. 7:**
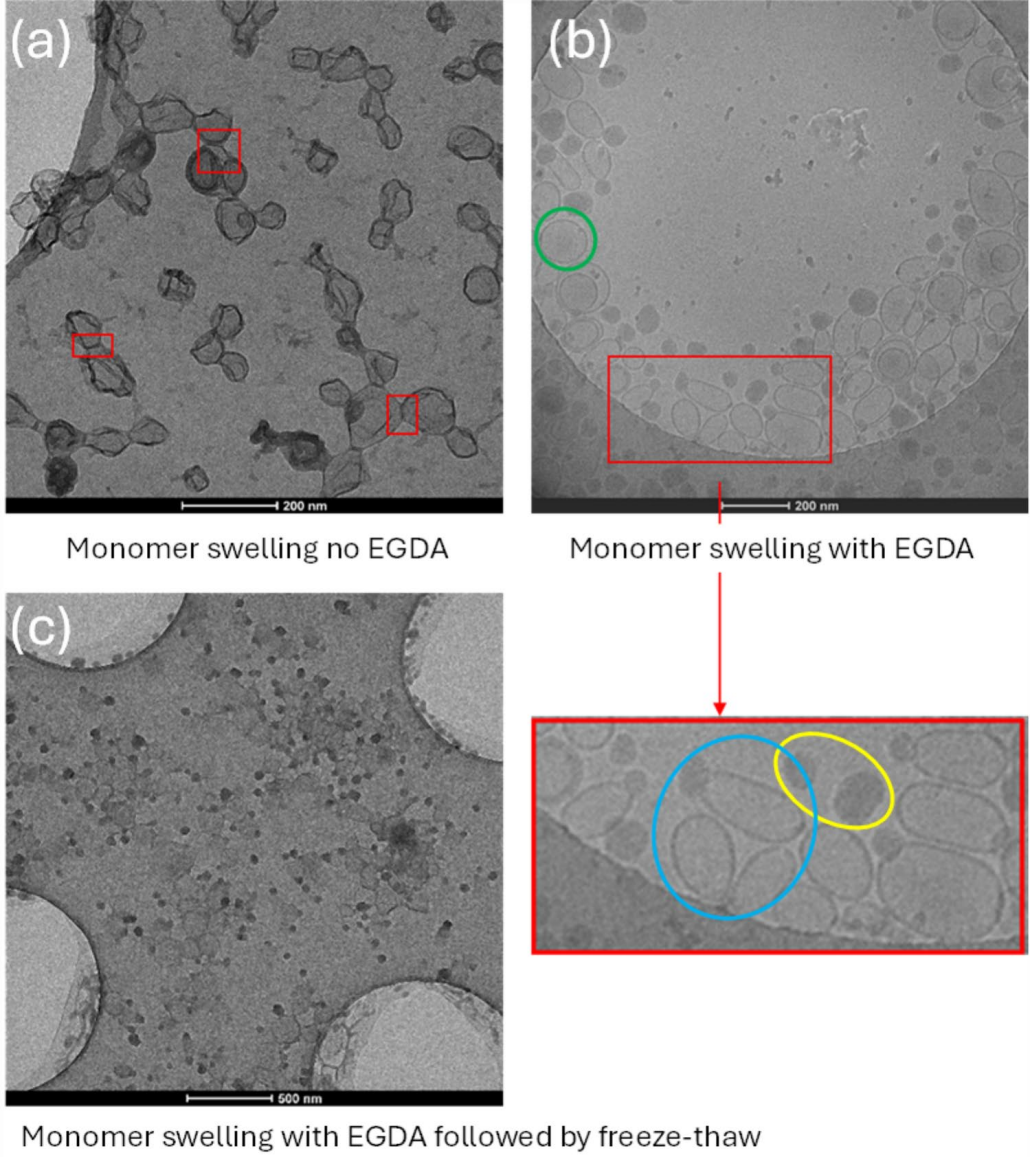
(**a**) Nanocapsules prepared via monomer swelling without EGDA, inter-nanocapsule links (highlighted in red frames). The polymeric shell appeared to be wrinkly instead of smooth, possibly due to the lack of cross-linker and the bulk addition of methyl acrylate. (**b**) Nanocapsules prepared via monomer swelling with EGDA, eliminated the rough shell but protrusion structures appeared possibly due to the bulk addition of the monomers in the lipid bilayer causing phase separation between the formed polymer and liposome bilayer, evidence of elongated nanocapsules highlighted in the red frame. Examples of solid nanoparticles were highlighted in yellow, spherical nanocapsules were highlighted in green, elongated nanocapsules with a solid cap were highlighted in blue (**c**) Re-freeze-thawed nanocapsules from Fig. 7b, which did not re-form any drug nanocrystals, presumed due to dissolution and leakage of drug from the liposomes during polymerization

The first polymerization was performed by adding methyl acrylate (MA) only (no EGDA) via monomer swelling. Cryo-TEM micrographs (Fig. 7a) showed nanocapsules with empty interior space were formed, with much thicker shells compared to the thin bilayer of liposomes. In the previous step, the adsorption of RAFT oligomers onto the liposomes could not be unequivocally confirmed by visualising with cryo-TEM, due to the small increment of shell thickness post-adsorption. However, the formation of the thick shells post-polymerization confirms that adsorption occurred and was able to drive the polymerization reaction on the surface of the elongated liposome templates. Undesirable free radical polymerization in the aqueous phase would have been evidenced by the presence of solid polymeric nanoparticles.

After polymerization, the ciprofloxacin drug nanocrystals had disappeared (Fig. 7a). The loss of ciprofloxacin from the capsules might be due to the high polymerization temperature. The phospholipid component in the lipid bilayer, HSPC, has a transition temperature of 55 °C [39]. Hence, at a reaction temperature of 65 °C, the lipid bilayer is expected to become permeable allowing drug to diffuse out of the liposome core. It might also be due to the higher solubility of ciprofloxacin at higher temperature driving dissolution. In future studies, a water-soluble initiator, such as VA-044, with a lower 10-h half-life temperature at 44 °C instead of 69 °C for V-501 could be used to decrease the reaction temperature to keep the ciprofloxacin in crystallized form and consequently maintain the elongated shape of the nanocapsules after polymerization. The elongated shape of the templates with RAFT oligomer adsorbed to their surface was not maintained after polymerization but the morphology of the resulting nanocapsules were not perfectly spherical either. They appeared to have a small degree of coupling between individual nanocapsules, and the polymeric shell was uneven instead of smooth. It is likely that chain-extension occurred between two templates creating the inter-nanocapsule bonds. Similar morphologies were observed in nanocapsules formed with high concentration of MA and low concentration of EGDA by Rusli et al. [12].

In the second system, a chain-extension polymerization with a cross-linker, adding EGDA eliminated the inter-nanocapsule bonds and the uneven shell (Fig. 7b). Three different types of nanoparticles were observed using MA and EGDA in combination with monomer swelling, including, (1) solid polymeric nanoparticles (circled in yellow), (2) spherical nanocapsules (circled in green) and (3) elongated nanocapsules with a solid (protruded) polymeric cap on one side of the nanocapsules (circled in blue). Spherical protruded nanocapsules have been reported in some literature while the kinetics of RAFT polymerisation was not well controlled with the choice of monomers [40, 41], but elongated nanocapsules using soft templates have remained elusive. Figure 7b illustrates that elongated nanocapsules were produced, even in the absence of residual internal drug nanocrystals. The fast radical polymerization in the lipid bilayer is proposed to maintain the elongated shape before the disappearance of the drug nanocrystals. Calculations from five cryo-TEM micrographs determined that approximately 15% of the nanocapsules were elongated.

As discussed earlier, the disappearance of drug nanocrystals might be due to the dissolution of drug inside the interior of the capsule, and/or leakage through the membrane. To determine whether drug remained inside the capsules in a saturated state, an attempt to re-crystallize the ciprofloxacin (Fig. 7c) by re-freezing the polymerized nanocapsules in liquid nitrogen and thawed in room temperature was carried out. However, no drug nanocrystals re-formed inside the polymeric nanocapsules, indicating that leakage of drug had most likely occurred, with any residual drug concentration below the level required for nanocrystal formation. This indicated a need to optimize the conditions for polymerization. The class of initiator and the corresponding reaction temperature is likely key if retention of nanocrystals is to be performed.

In the literature, two slightly different types of nanocapsules with a solid cap were observed, namely the parachute structure and the protrusion structure. With the parachute structure, the solid cap was not part of the shell of the nanocapsules or vesicles and a complete circular shell of the hollow nanocapsules or vesicles could be observed [12]. With the protrusion structure, the solid cap was part of the shell and the vesicle was wrapped in a polymeric layer [12]. In this case, the polymer cap appeared to be part of the nanocapsules, so were classified as a protrusion structure.This in turn indicated the successful adsorption of RAFT oligomers on the surface driving the polymerization reaction to form a polymeric layer around the liposome templates. It was demonstrated by Hubert et al*.* that the formation of a solid cap in polymer-vesicle colloids was due to the intrinsic incompatibility of the formed polymer chain with the bilayer [17]. In this case, due to the monomer swelling addition profile, it is possible that monomers accumulate in the hydrophobic bilayer of the liposome templates. Due to the high local concentration of monomers, an emulsion polymerization occurred either in the free solution or in a monomer droplet. This results in the formation of long polymeric chains causing phase separation from the liposome templates. In summary, the solid nanoparticles and the solid cap on the side of the nanocapsules are the result of a degree of uncontrolled free-radical polymerization.

### Effect of monomer addition method on nanocapsule morphology

**Fig. 8:**
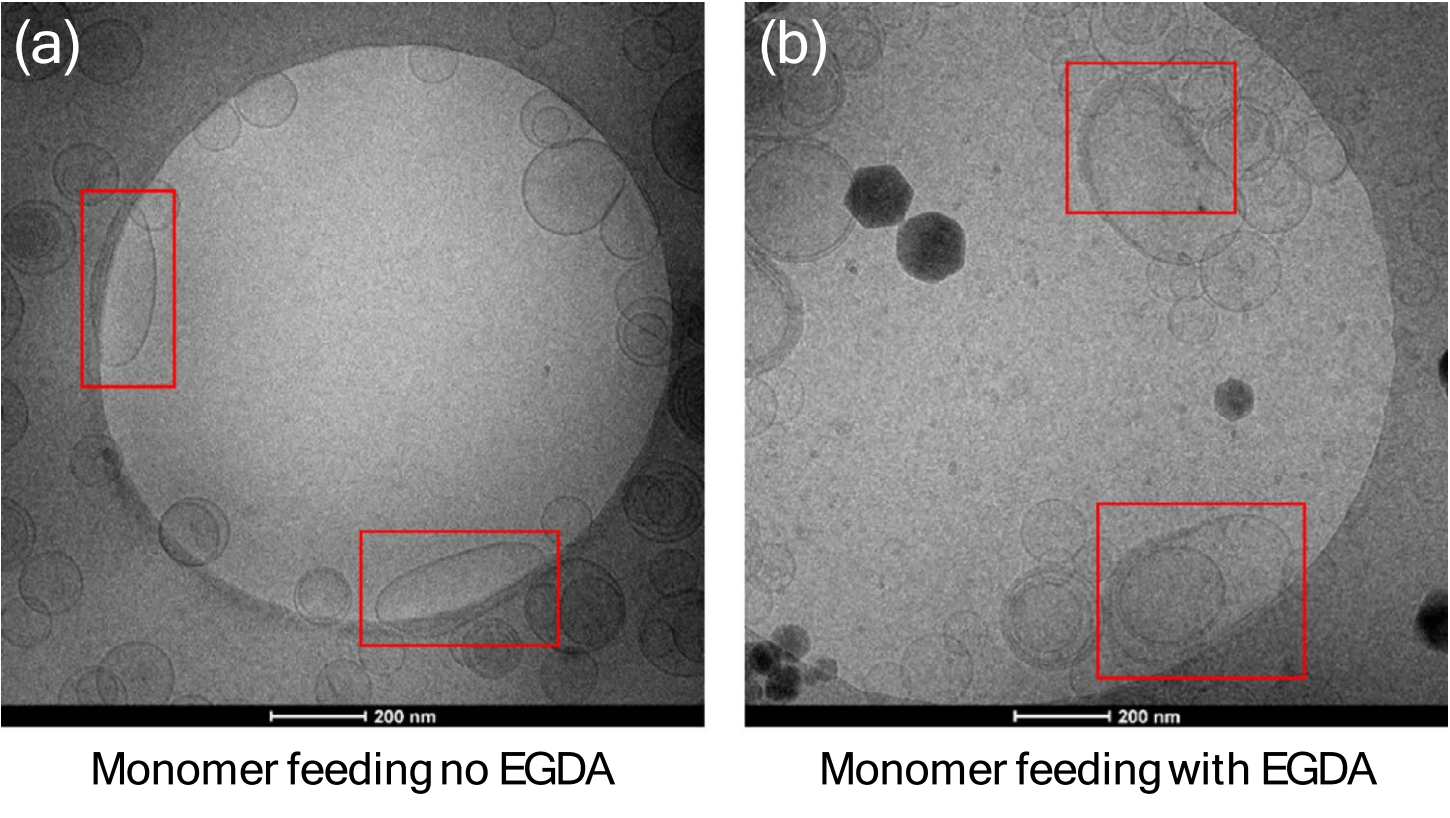
Polymerized nanocapsules prepared (**a**) without EGDA and (**b**) with EGDA by monomer feeding. Elimination of the protrusion cap was observed, but the population of elongated nanocapsules decreased, indicating the importance of the balance between polymerization kinetics and the disappearance of the ciprofloxacin nanocrystals. The shell was thinner compared to when the bulk addition method was used. Elongated nanocapsules are highlighted in red frames

To prepare elongated nanocapsules without the protrusion cap, capsules were also prepared by slow addition of either MA, or a mixture of MA and EGDA. Moving from monomer swelling to feeding resulted in an absence of protrusion caps both without (Fig. 8a) and with EGDA (Fig. 8b). This indicates that other processes occurred with the swelling addition method, such as emulsion polymerization in the aqueous phase or in monomer droplets. Which did not occur with monomer feeding, consistent with previous observations by Rusli et al*.* [12]. However, the population of elongated nanocapsules was dramatically reduced compared to monomer swelling. This was possibly because RAFT controlled polymerization could only occur on the surface of the liposome templates where the oligomers are, while other processes such as emulsion polymerization could happen in the free solution with monomer droplets. The amount of oligomer adsorbed onto the surface was limited as described previously. The dissolution kinetics of the drug nanocrystal have not been previously measured in this medium and are out of the scope of the study. However, the dissolution of drug may have been initiated when the temperature was increased, at an earlier time than the onset of the polymerization reaction. In addition, the temperature needs to reach a certain value for initiator to produce sufficient radicals to start the polymerization. Thus, if the required reaction temperature is higher than the phase transition temperature of the main lipid, HSPC, the drug will start to dissolve. Then the leaky lipid membrane allowed the release of drug from the liposome along the concentration gradient prior to polymerization. If the polymeric shell was able to form before the disappearance of drug nanocrystals, the elongated shape could be retained.

### Quantitative analysis of the thickness of the shell

**Fig. 9:**
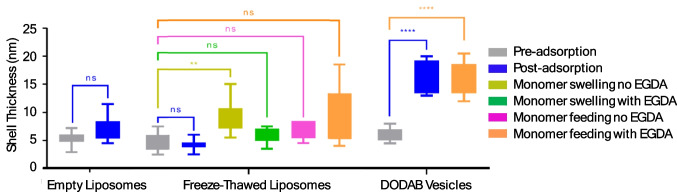
Quantitative analysis of shell thickness pre- and post-adsorption of the RAFT oligomer, and after polymerization with different reaction conditions. The shell was significantly thicker than the pre-polymerization case only for spherical nanocapsules prepared with DODAB vesicles and nanocapsules prepared with the bulk addition method without EGDA. The boxplot was plotted with the minimum and the maximum values (n = 10). If P > 0.05, not significant (ns); **P < 0.01 ****P < 0.0001 (t-test; two-tailed). P stands for probability and measures how likely it is that any observed difference between groups is due to chance

To further confirm the formation of a polymeric layer on the surface of liposome templates, the thickness of the shells was measured from cryo-TEM images (Fig. 9). Quantitative analysis was performed for each sample with five individual nanoparticles of various size from different cryo-TEM micrographs. The thickness of the shell was measured and calculated with the method described in the “Visualization with Cryo-TEM” section. As previously described, spherical nanocapsules formed by DODAB vesicle templation were used as a comparison. For DODAB vesicles, the electrostatic interactions between the positively charged DODAB vesicles and the negatively charged RAFT oligomers provided a driving force for oligomer adsorption. Therefore, a very significant increase of shell thickness was shown after adsorption. For liposome templates, only the nanocapsules performed with monomer swelling without EGDA showed significance in the increment of shell thickness. Nanocapsules prepared by other methods did not show any significant increase in the shell thickness. It could not be confirmed with the quantitative analysis whether most of the polymeric shells were significantly thicker than the liposome templates, due to the limitation of magnification of cryo-TEM and the method of measurement. However, as discussed previously, according to the hollow feature of the polymerized nanocapsules, the polymeric layer formed. If adsorption was unsuccessful, the polymer would have appeared as solid nanoparticles instead of hollow nanoparticles. The quantitative measurement, however, was still of great significance to assist visual observations of cryo-TEM micrographs. Such a measurement was rarely performed previously but should be used more frequently in future studies to make the full use of microscopy images.

### Verification of the presence of a polymeric shell on the particle surface

Due to the thin polymeric layer on the surface of the liposome template, results from quantitative cryo-TEM were not significant to verify the existence of the shell. Therefore, another method was needed to confirm the successful polymerisation. The hypothesis of this final experiment was that upon mixing with surfactant (in this case Triton X-100), only lipid would be solubilized and not crosslinked polymer. The presence of intact particles using DLS was used as the measure of whether particles were retained after this treatment. Intact particles would be evident at ~ 150 nm, or if solubilized then only micelles ~ 10 nm would be present. DLS results showed that only the polymerized nanocapsules (both with and without EGDA) retained a peak in the size distribution at 150–200 nm (Table 2, green highlight), which means only the formed polymeric layer was able to resist the membrane-lysing effect of Triton X-100 solution. The peak in the size distribution for pure Triton X-100 was at approximately 10 nm, confirming the appearance of micellar peak in most samples. DLS measurement showed only micelles present for empty, FT, and elongated liposomes exposed to the oligomer samples supporting the hypothesis and confirming the formation of a polymer capsule only for polymerized systems.

**Table 2:**
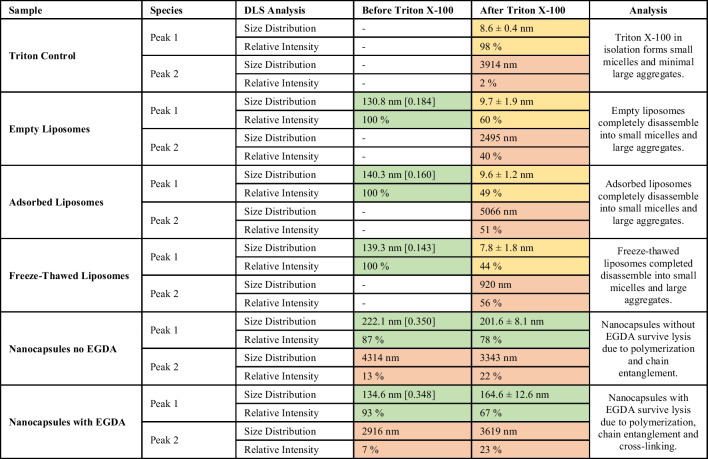
Size distribution of empty liposomes, freeze-thawed liposomes, liposomes with adsorbed oligomer, and polymerized liposomes with and without EGDA. For the nanocapsules presented in the table, monomers were added all at once (swelling method). DLS analysis (intensity average) presented before and after the addition of Triton X-100. Average size in nanometer and [polydispersity index] is presented when Peak 1 forms a majority of the sample. For data after the addition of Triton, due to the presence of two (relatively equal) size distribution peaks, the polydispersity index was not quoted because it reflected the polydispersity of the whole sample. Instead, the standard deviation of each individual peak was presented. For ease of presentation, distribution peaks representing nanocapsules are highlighted in green, micellar peaks arising from lysed liposomes and/or excess Triton are highlighted in yellow and large aggregate peaks (greater than 1000 nm) are highlighted in red

In this study, it was proven that liposomes could be used as templates for the formation of nanocapsules via the vesicle templation route. The previously reported formation of spherical nanocapsules via DODAB vesicle templation relied on using a 0.5 mM NaCl solution upon hydration [12], which limits its application in drug delivery where simulated biological fluid conditions, such as PBS buffer with a much higher ionic strength is required. Natural lipid materials are also more biocompatible than surfactant materials. Therefore, this study opened opportunities to use liposomes to encapsulate pharmaceutical actives under biologically compatible conditions, then serve as the template, to prepare nanocapsules via the vesicle templating route making use of the cross-linked polymeric shell to provide more protection for the drugs.

The successful adsorption of RAFT oligomers on the surface of elongated liposomes could be confirmed by the formation of hollow nanocapsules instead of solid polymer nanoparticles, but the amount of RAFT oligomer successfully adsorbed requires optimization. Electrostatic interactions play an important role in the formation of hollow spherical nanocapsules with DODAB vesicle templation. It could be feasible to add a small amount of DODAB molecules within the lipid formulation to provide some positive surface charge for improved adsorption. Elongated liposomes encapsulating ciprofloxacin nanocrystals functionalized with azide functional group have been previously developed [32] and RAFT oligomers functionalized with the complementary alkyne group could also be synthesized [42], providing the opportunity to use ‘click’ chemistry reaction instead of electrostatic interaction as the adsorption mechanism.

The existence of the polymeric shell on the surface of the liposome template was verified by using Triton X-100 to lyse the lipid bilayer first and then DLS to confirm the existence of nanoparticles post-lysis. The different methods of polymerisation produced nanocapsules with varying morphologies. The reaction kinetics of the swelling method was less controlled than that of the feeding method, however the percentage of elongated nanocapsules at the end of the polymerisation from the swelling experiment was significantly greater than that from the feeding method. To increase the percentage of elongated nanocapsules, preserving the drug nanocrystals inside the liposome templates throughout the whole polymerisation procedure is the key. Methods such as using a lower temperature initiator such as VA-044 could be used in future investigations. Using UV-initiated polymerisation could also potentially tackle the drug loss issue, however, UV may accelerate the degradation of many drugs including antibiotics and chemotherapy drugs. Hence, using VA-044 is a priority over conducting UV-initiated polymerisation in future investigations.

If drug nanocrystals can be preserved in future investigations, the cross-linked polymeric shell will play a role in achieving delayed release. Compared to self-assembled structures such as liposomes, cross-linked polymer is much more resistant to disassembly factors such as dilution upon intravenous administration and sheer stress from blood circulation. The release kinetics of the drug is anticipated to be dependent on both the dissolution of drug nanocrystal and diffusion across the capsule wall, as well as the digestion of the polymeric shell by enzymes in the body which breaks down chemical bonds such as ester or carbon bonds. Hence, a high payload could be achieved with the loading of high concentration of drug as nanocrystals and a sustained release is achieved due to the slow digestion process of the polymeric shell, with the goal to significantly decrease the frequency of administration in clinical practice. It is possible in this situation that if the barrier remains intact, that drug release could be linear, as it diffuses across the capsule wall from a constant supply (the saturated solution surrounding the nanocrystal) until it runs out of crystallized drug.

## Conclusions

The study presents a proof-of-concept synthetic route for elongated nanocapsules via elongated liposome templated polymerization (ELTP). Elongated liposomes were prepared via drug loading and freeze-thawing, affording encapsulated drug nanocrystals. Conversion into elongated liposome templates was followed by the adsorption of RAFT oligomers, which is supported by DLS and cryo-TEM. Successful chain-extension polymerization was followed by monomer conversion via GC–MS, cryo-TEM of the resulting morphology and lysis experiments with surfactant further confirmed the presence of a polymeric shell using DLS. The morphology of the nanocapsules is dependent on the presence of cross-linker and monomer addition profiles. After polymerization, the internal drug nanocrystals disappeared, possibly due to high temperature used in the reaction and phase transition of the main lipid component causing drug dissolution and leakage from the capsule. This initial study confirmed that ELTP is an intriguing platform approach, which can be further improved with the aim of increasing drug encapsulation, modulating shell thickness, controlling nanocapsule morphology and optimizing nanocapsule yield. Comparison to the previous reported DODAB vesicle templated approach confirmed that RAFT oligomer adsorption is the pivotal step in the use of soft (non-sacrificial) templates. Overall, this methodology holds great promise as it combines biocompatible liposomes, synthetic RAFT-mediated polymers which can readily incorporate stimuli-responsive behaviour and the elongated morphology which can be utilized to improve circulation time and biodistribution.

## Supplementary Information

Below is the link to the electronic supplementary material.

Supplementary file1 (DOCX 1187 KB)

## Data Availability

All data are available on request to the corresponding author.

## Declarations

### Supporting information

RAFT Oligo characterization (^1^H NMR, size exclusion chromatography), polymerization kinetics (GC–MS) and measurement of shell thickness via FIJI software.
