# Supplementary material for: Asymmetric nanocapsules via elongated liposome templated polymerization (ELTP) mediated by RAFT polymerization
Source: Drug Deliv Transl Res. 2025 Mar 6;15(9):3252–67. doi: 10.1007/s13346-025-01805-z (PMC12350517; doi:10.1007/s13346-025-01805-z)
Supplement: Supplementary file 1 — Supplementary file1 (DOCX 1187 KB) [file 13346_2025_1805_MOESM1_ESM.docx]

**Electronic Supplementary Information**

Asymmetric Nanocapsules via Elongated Liposome Templated Polymerization (ELTP) Mediated by RAFT Polymerization

Yunxin Xiao^1,2^, Alexander W. Jackson^3^, Angel Tan^1,2^, John F. Quinn^1,2,4^, Simon Crawford^5^, and Ben J. Boyd^1,2,6,*^

*^1^Drug Delivery, Disposition and Dynamics, Monash Institute of Pharmaceutical Sciences, Monash University Parkville Campus, 381 Royal Parade, Parkville, VIC, 3052, Australia.*

*^2^ARC Centre of Excellence in Convergent Bio-Nano Science and Technology, Monash University Parkville Campus, 381 Royal Parade, Parkville, VIC, 3052, Australia.*

*^3^Institute of Sustainability for Chemicals, Energy and Environment (ISCE^2^), Agency for Science, Technology and Research (A*STAR), 1 Pesek Road, Jurong Island, 627833, Singapore.*

*^4^Department of Chemical Engineering, Faculty of Engineering, Monash University, Clayton, VIC 3800, Australia.*

*^5^Ramaciotti Centre for Cryo-Electron Microscopy,* *Monash University Clayton Campus, 15 Innovation Walk, Wellington Road, VIC 3800, Australia.*

*^6^Department of Pharmacy, University of Copenhagen, 2100 Copenhagen, Denmark.*

Email: Ben.Boyd@moansh.edu (Ben J. Boyd)

**Supplementary Information 1: Preparation and Characterisation of RAFT Oligomer**


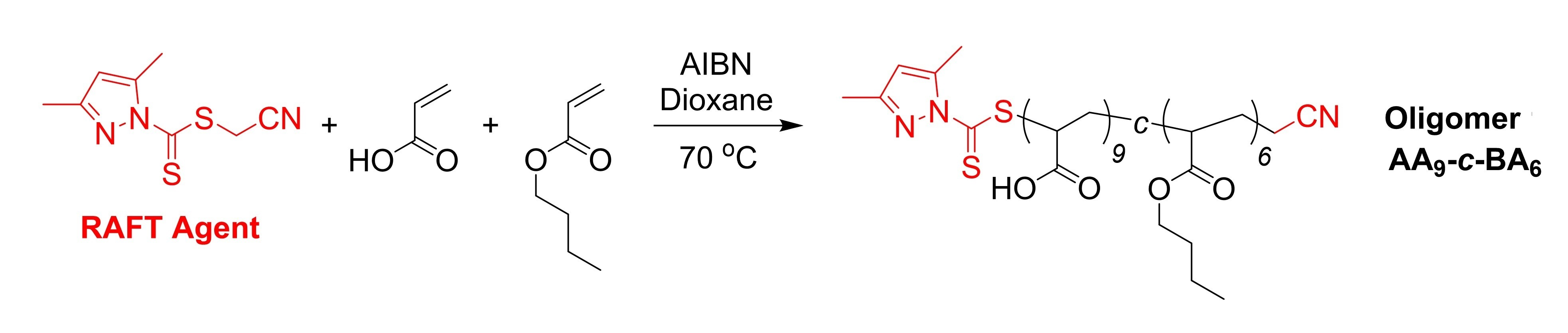


^1^H NMR spectra were recorded on a Bruker 400 Ultra Shield spectrometer in acetone-*d*_6_. Size exclusion chromatography (SEC) was conducted on a Viscotek TDAmax consisting of a GPCmax integrated solvent and sample delivery module, a TDA 302 Triple Detector Array, and OmniSEC software. 2 x PLgel 5 µm Mixed-C (200-2,000,000) columns were applied in sequence for separation. THF was used as the eluent at 1.0 mL/ min with column and detector temperature at 30 °C, molecular weight values were determined against polystyrene standards.

^1^H NMR spectra (acetone-*d*_6_) of RAFT Oligomer (AA_9_-co-BA_6_).


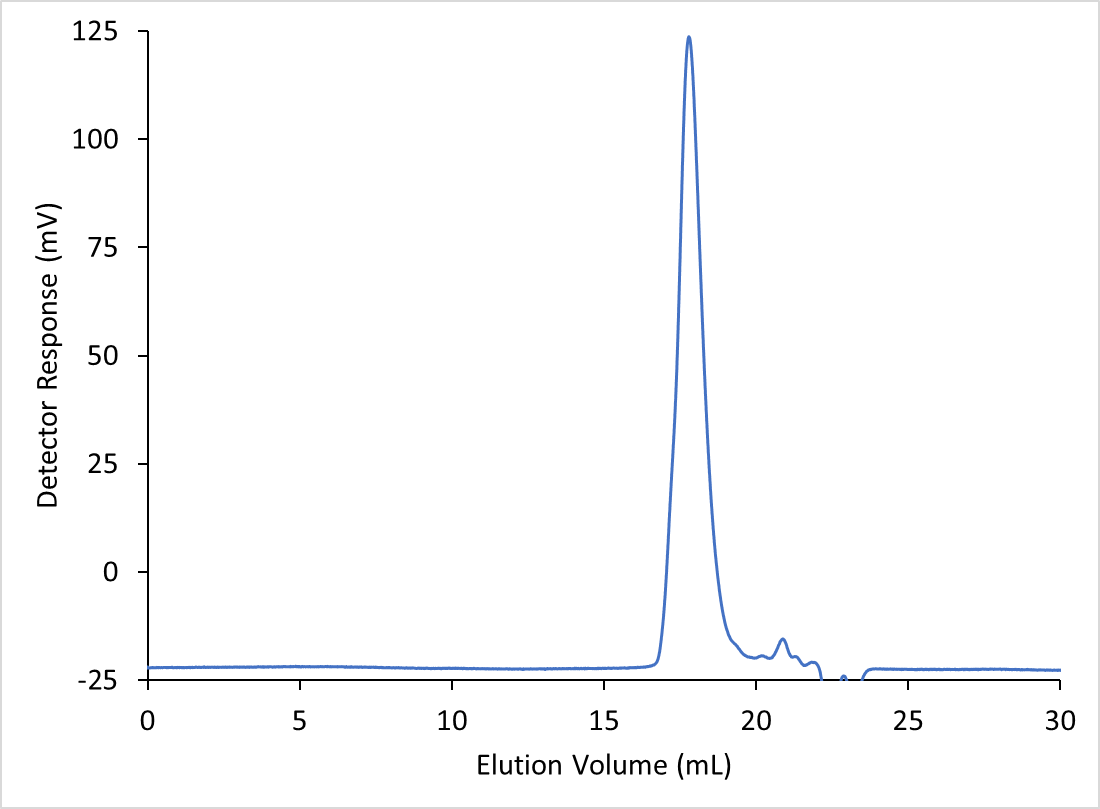


SEC trace of RAFT Oligomer (AA_9_-co-BA_6_). Molecular weight analysis: *M*_n_ = 1900 Da, *M*_w_ = 2600 Da, Dispersity (*Đ*_M_) = 1.37.

**Supplementary Information 2: GC-MS Standard Curves**

Service of the MS, change of GC column in between experiments would change the sensitivity of the detection. Therefore, two standard curves were generated along with samples run at two different times. The corresponding equation was used for each sample to calculate the amount of residual methyl acrylate (MA)


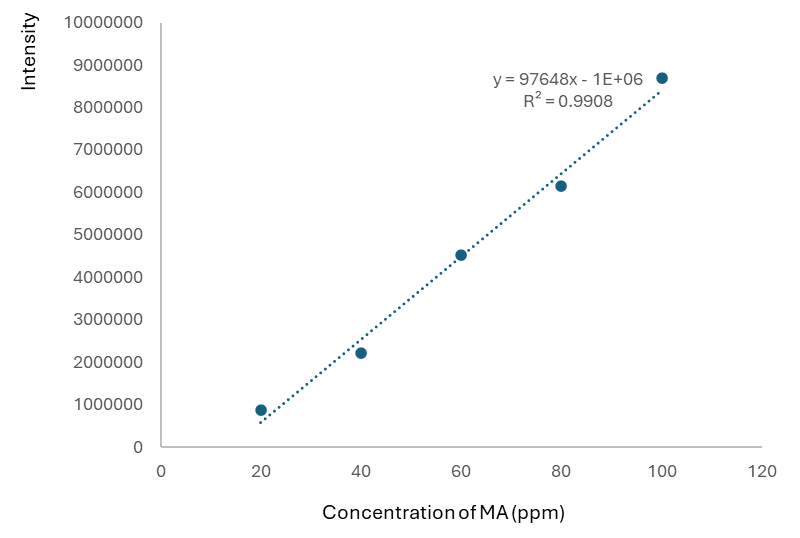


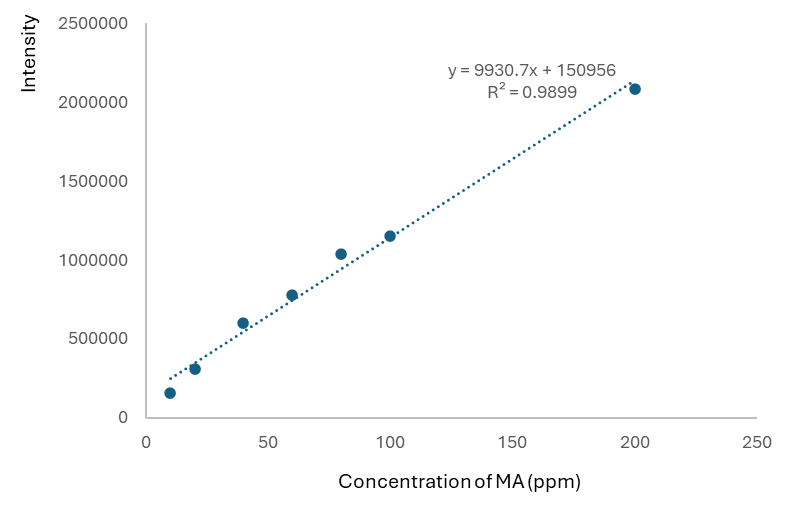


**Supplementary Information 3: GCMS Comparison Graphs and Mass Spectrum of Methyl Acrylate**

Comparison graph of GC chromatograms of standards for the first standard curve


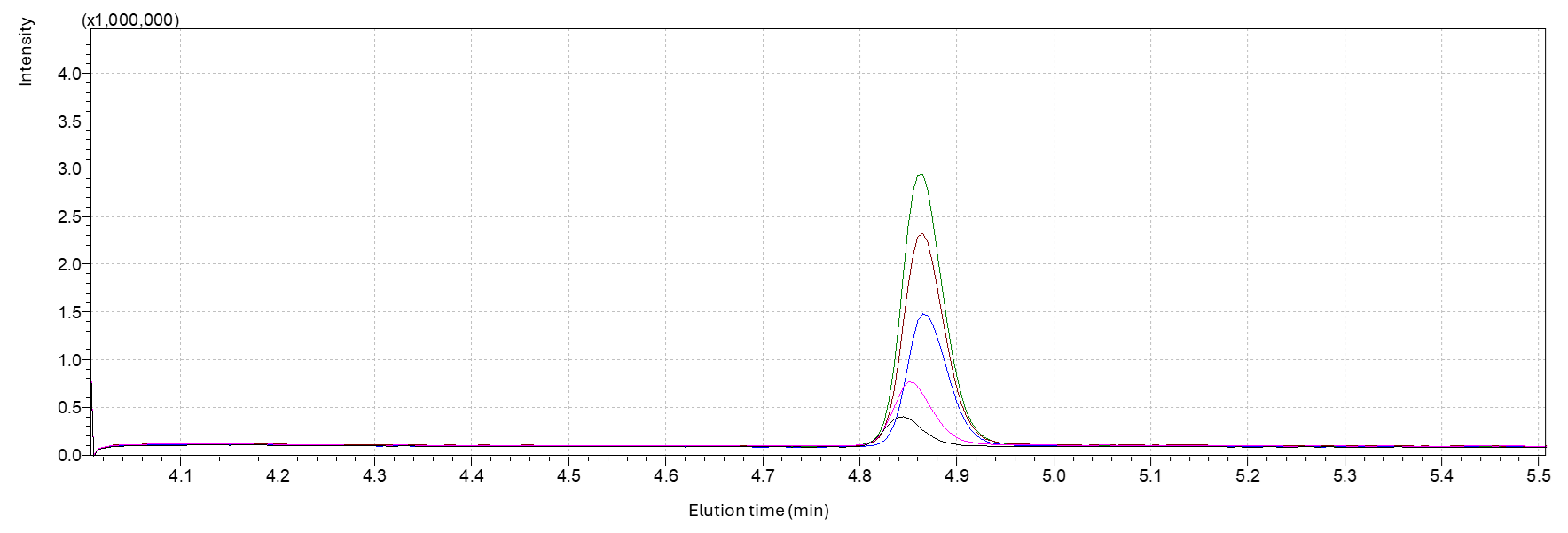


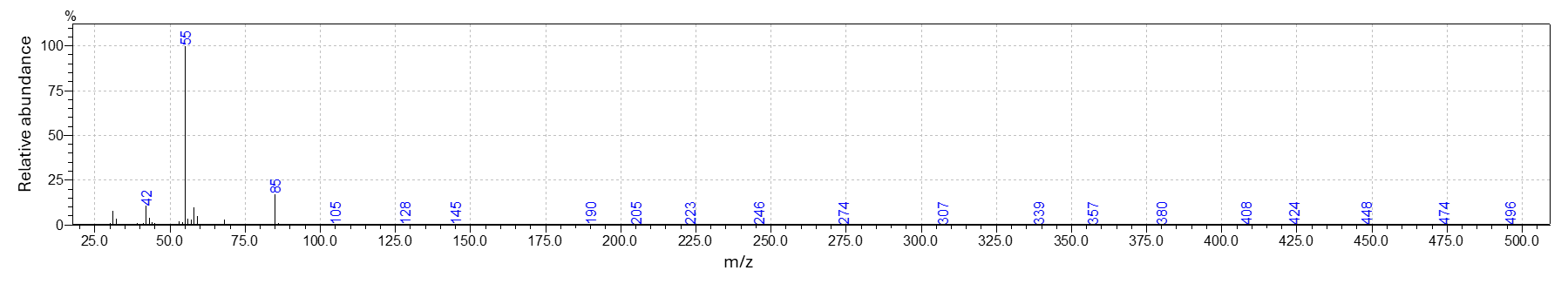


Comparison graph of GC chromatograms of standards for the second standard curve


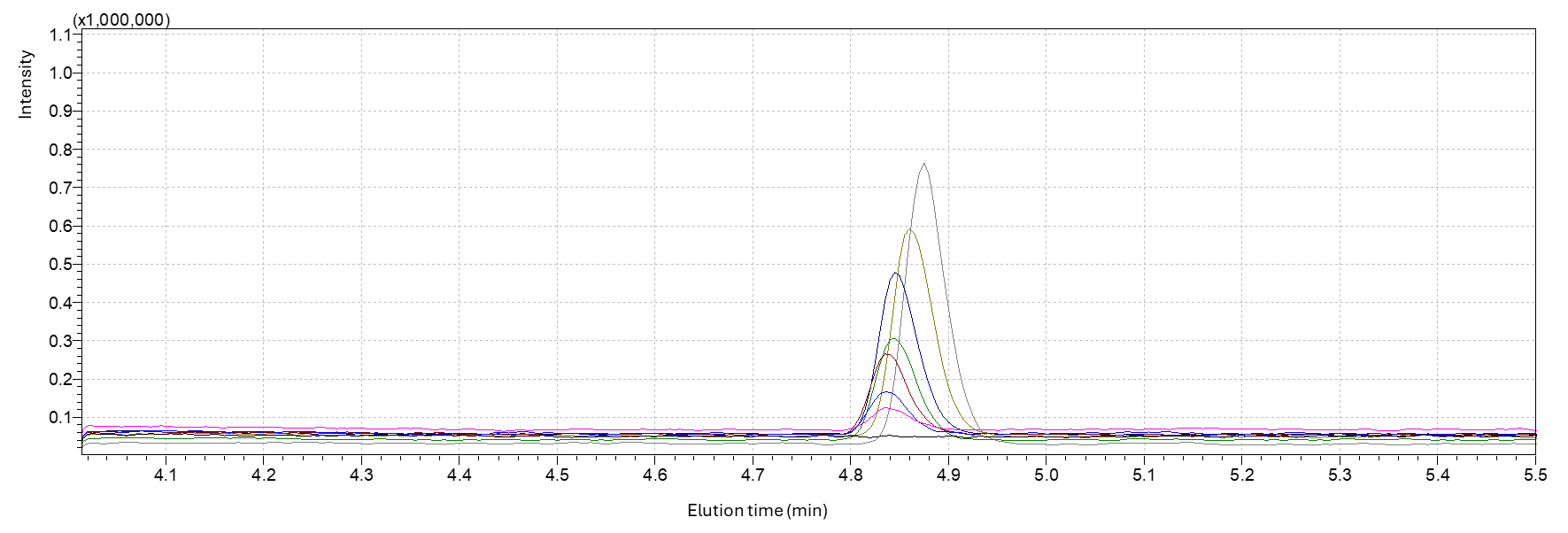


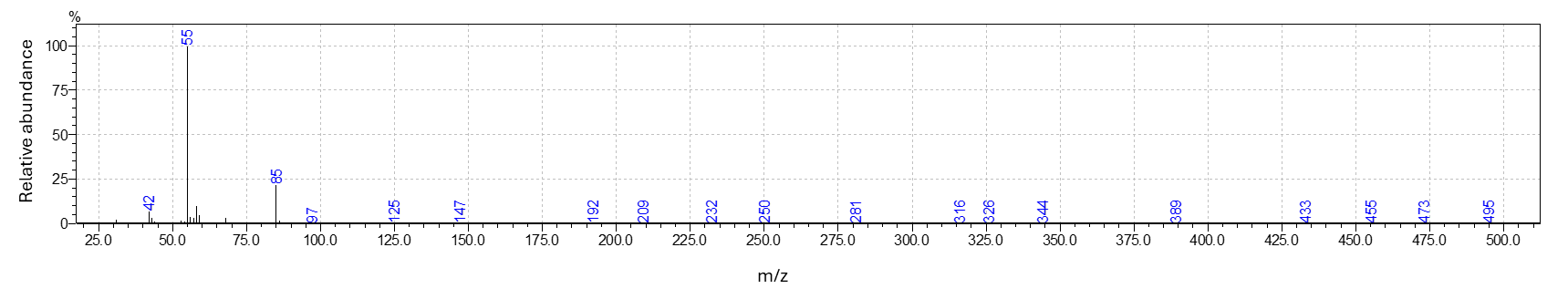


Sample: Swelling, without EGDA


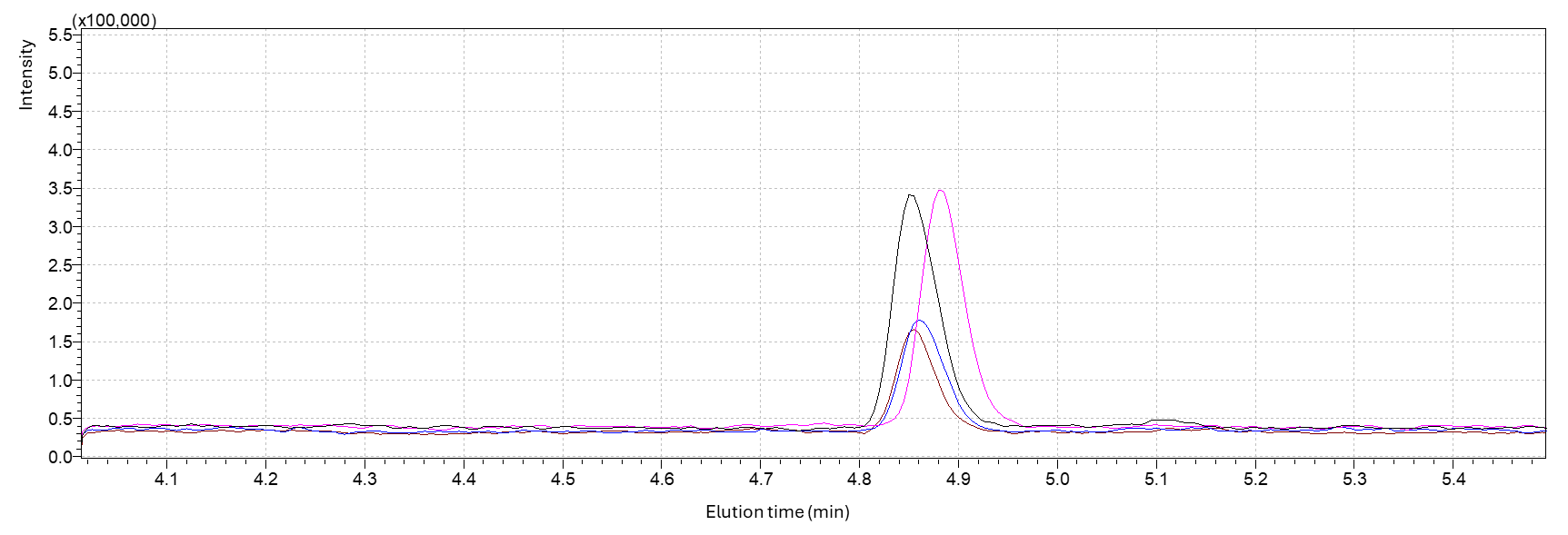


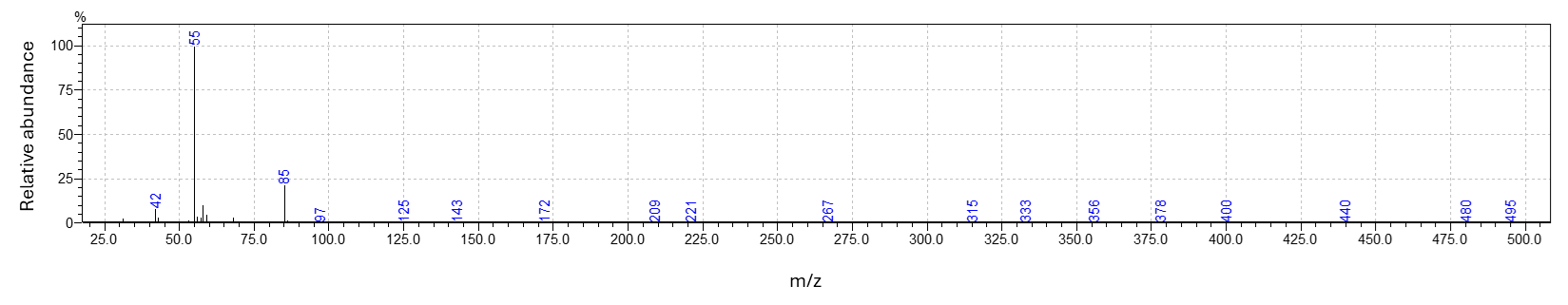


Sample: Swelling, with EGDA


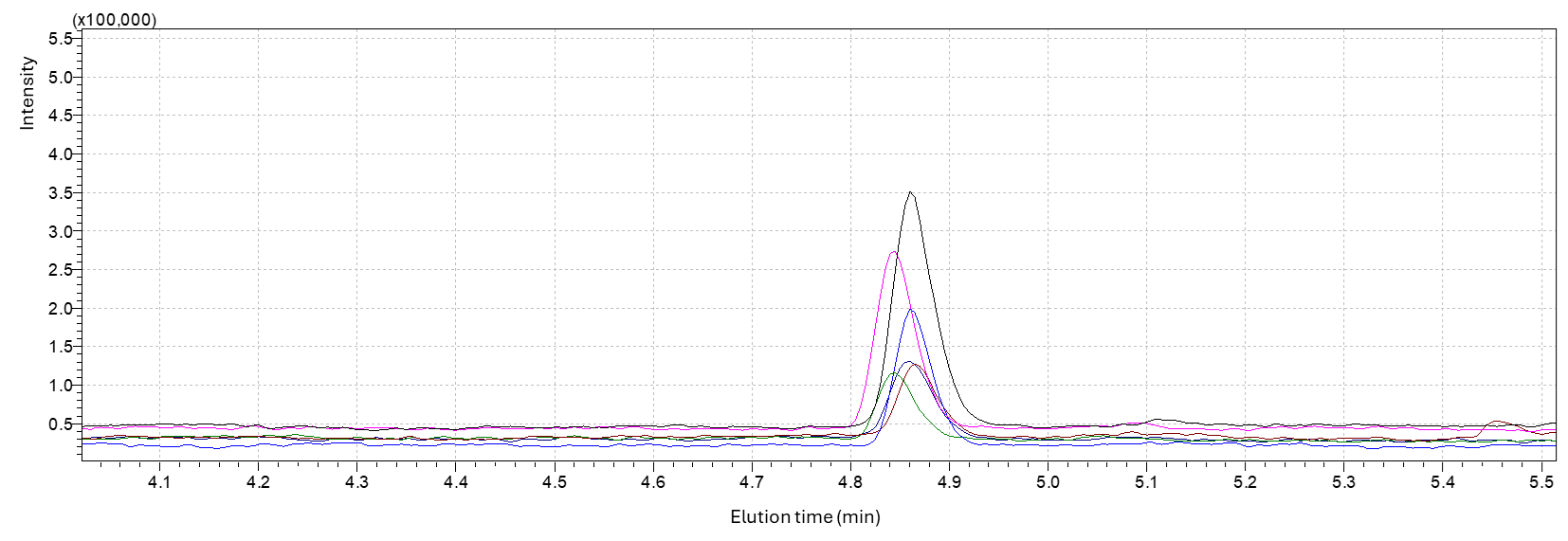


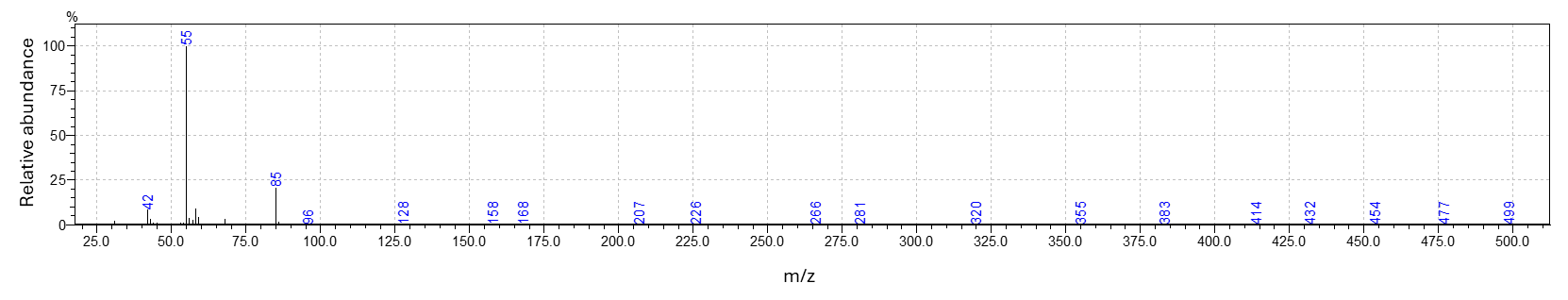


Sample: Feeding, without EGDA


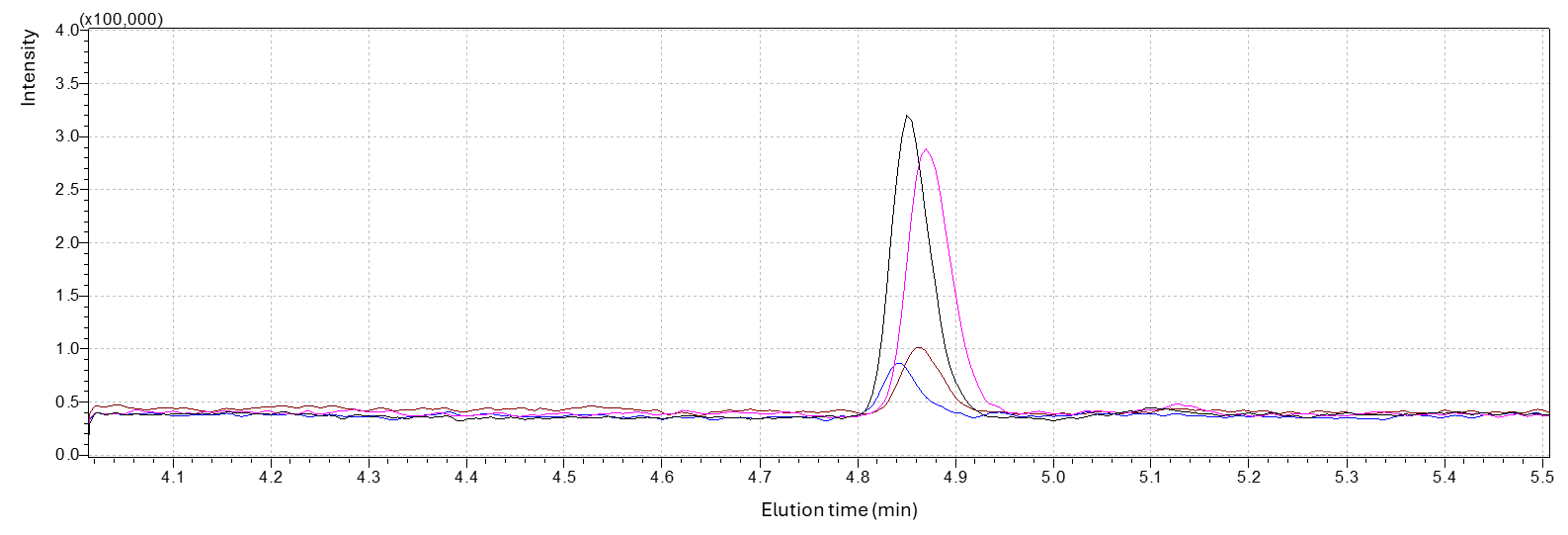


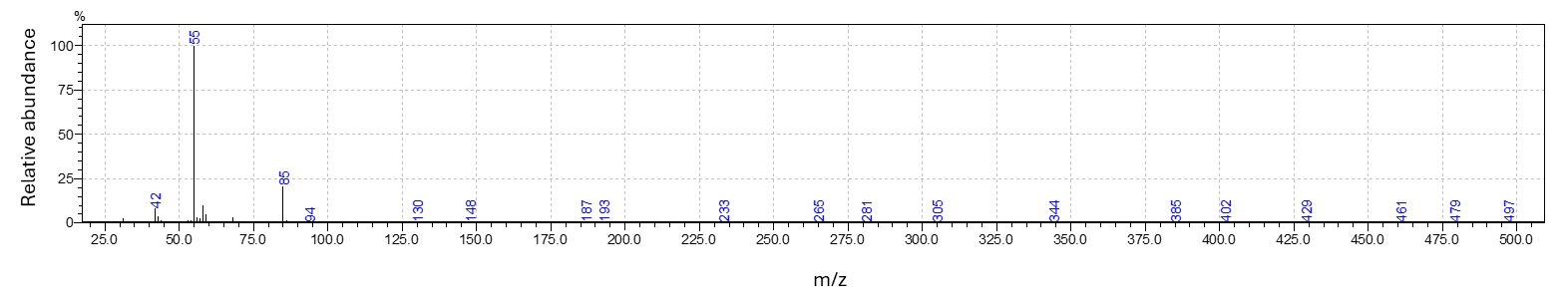


Sample: Feeding, with EGDA


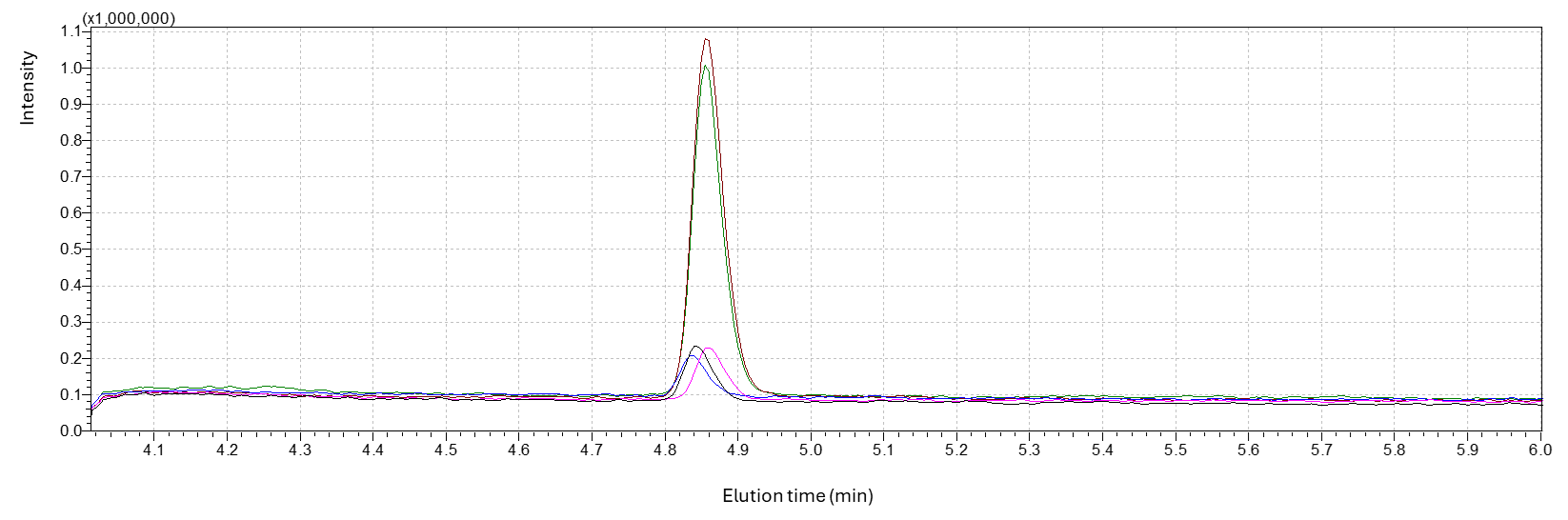


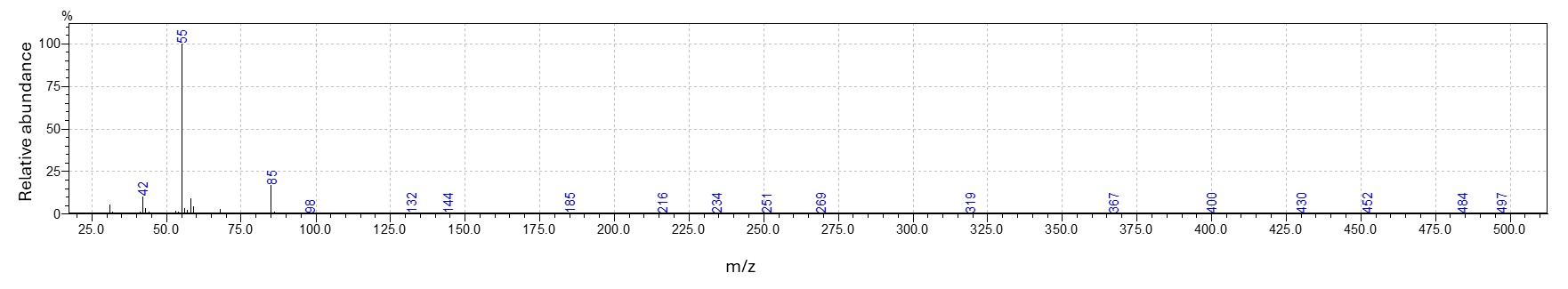


**Supplementary Information 4: Measurement of Shell Thickness using FIJI Software**


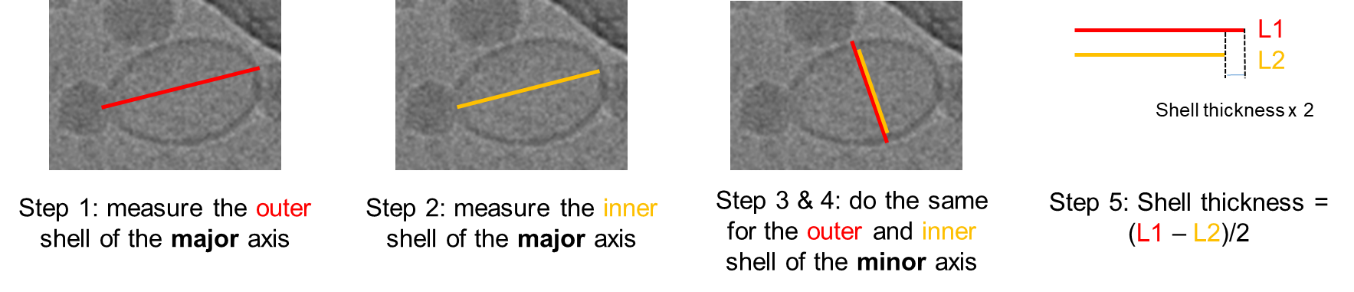


The shell thickness of the liposomes or nanocapsules were calculated from the measurements of the outer shell and the inner shell of both major and minor axis.
